# Supplementary material for: eIF4E-independent translation is largely eIF3d-dependent
Source: Nat Commun. 2024 Aug 6;15:6692. doi: 10.1038/s41467-024-51027-z (PMC11303786; doi:10.1038/s41467-024-51027-z)
Supplement: Supplementary file 1 — Supplementary Information [file 41467_2024_51027_MOESM1_ESM.pdf]

Supplementary Information

eIF4E-independent translation is largely eIF3d-dependent

Mykola Roiuk[1,2], Marilena Neff[1,2], and Aurelio A. Teleman[1, 2]

Contents

Supplementary Figures .... p. 2

Uncropped Immunoblots of Supplementary Figures p.42

References for Supplementary Information.. p. 60

Suppl. Figure 1

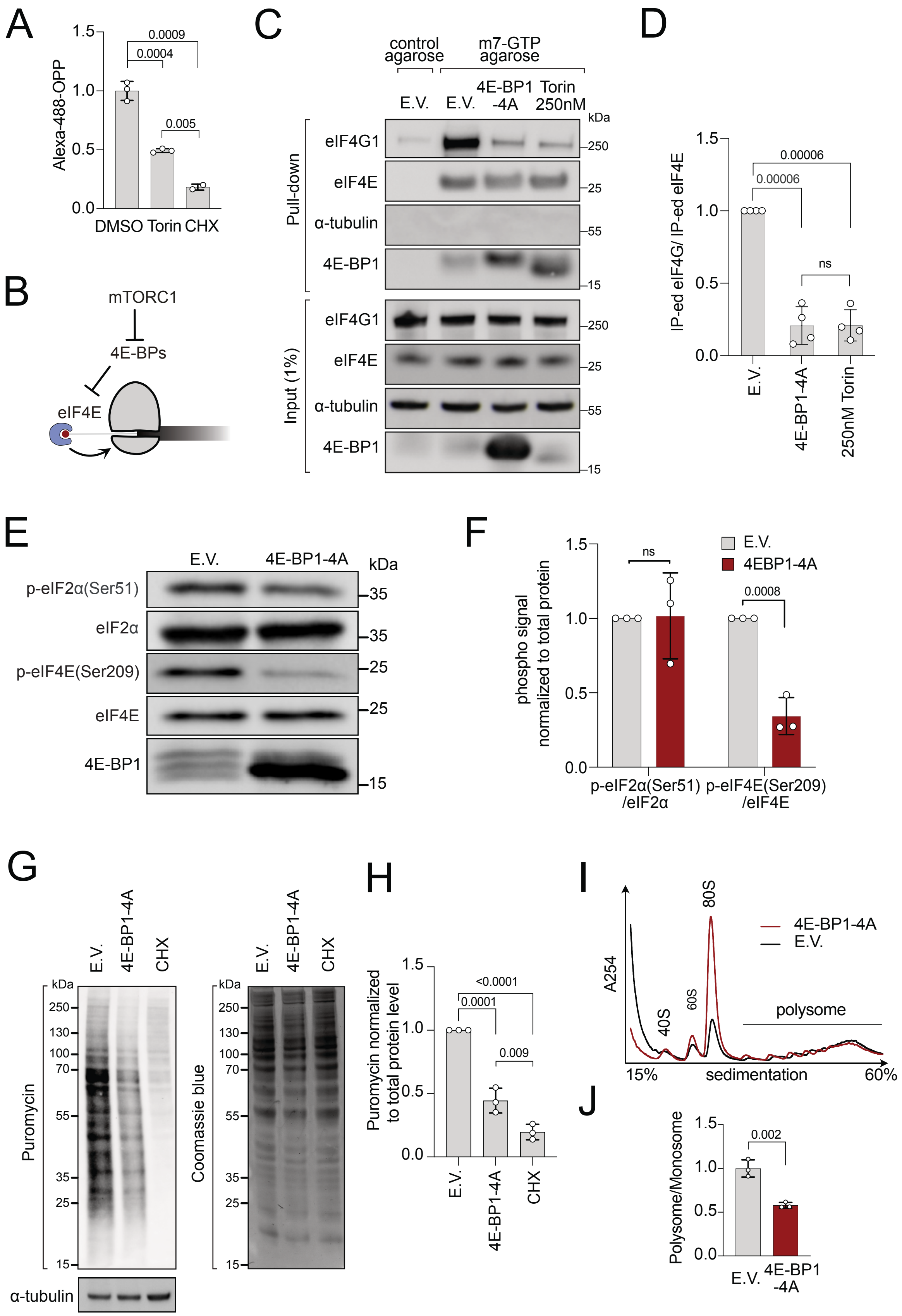

Suppl. Figure 1: mTORC1 inhibition does not completely block translation.

(A) mTOR inhibition reduces but does not completely block translation. Global protein synthesis was assessed by OPP incorporation in HeLa cells treated either with DMSO, 250nM Torin or 400μM cycloheximide (CHX). n=3 biological replicates, error bars=std. dev., significance by unpaired, two-sided, t-test.

(B) Schematic illustration of 4E-BPs action.

(C-D) 4E-BP1-4A disrupts eIF4G1 binding to eIF4E. m-7-GTP cap pull-down with lysates from HeLa cells overexpressing either 4E-BP1-4A or empty vector (E.V.). The effect of 4E-BP1-4A is comparable to 1 hour 250nM Torin treatment. (C) Representative blot. (D) Quantification of 4 independent experiments. Error bars = std. dev., significance by unpaired, two-sided, t-test adjusted for multiple testing. ns=not significant.

(E-F) 4E-BP1-4A expression does not induce eIF2alpha phosphorylation. Immunoblot of lysates from HeLa cells transfected to express 4E-BP1-4A or nothing as a control (empty vector, E.V.). (E) Representative immunoblot. (F) Quantification of 3 independent biological replicates. Error bars represent standard deviation. Significance by unpaired, two-sided, t-test. ns=not significant.

(G-H) 4E-BP1-4A overexpression blunts global translation. Cells overexpressing either 4E-BP1-4A or empty vector were treated with OPP for 1 hour. Global protein translation levels were assessed by immunoblotting against OPP, normalized to total protein assayed by Coomassie blue. (G) Representative blot. (H) Quantification of three independent replicates. n=3 biological replicates, error bars=std. dev., significance by one-way ANOVA.

(I-J) 4E-BP1-4A overexpression reduces the polysome-to-monosome ratio. Lysates of HeLa cells overexpressing either 4E-BP1-4A or vector control were separated on a sucrose gradient. (I) Representative polysome gradient. (J) Quantification of polysome to monosome for 3 independent experiments. error bars=std. dev., significance by unpaired, two-sided, t-test.

Suppl. Figure 2

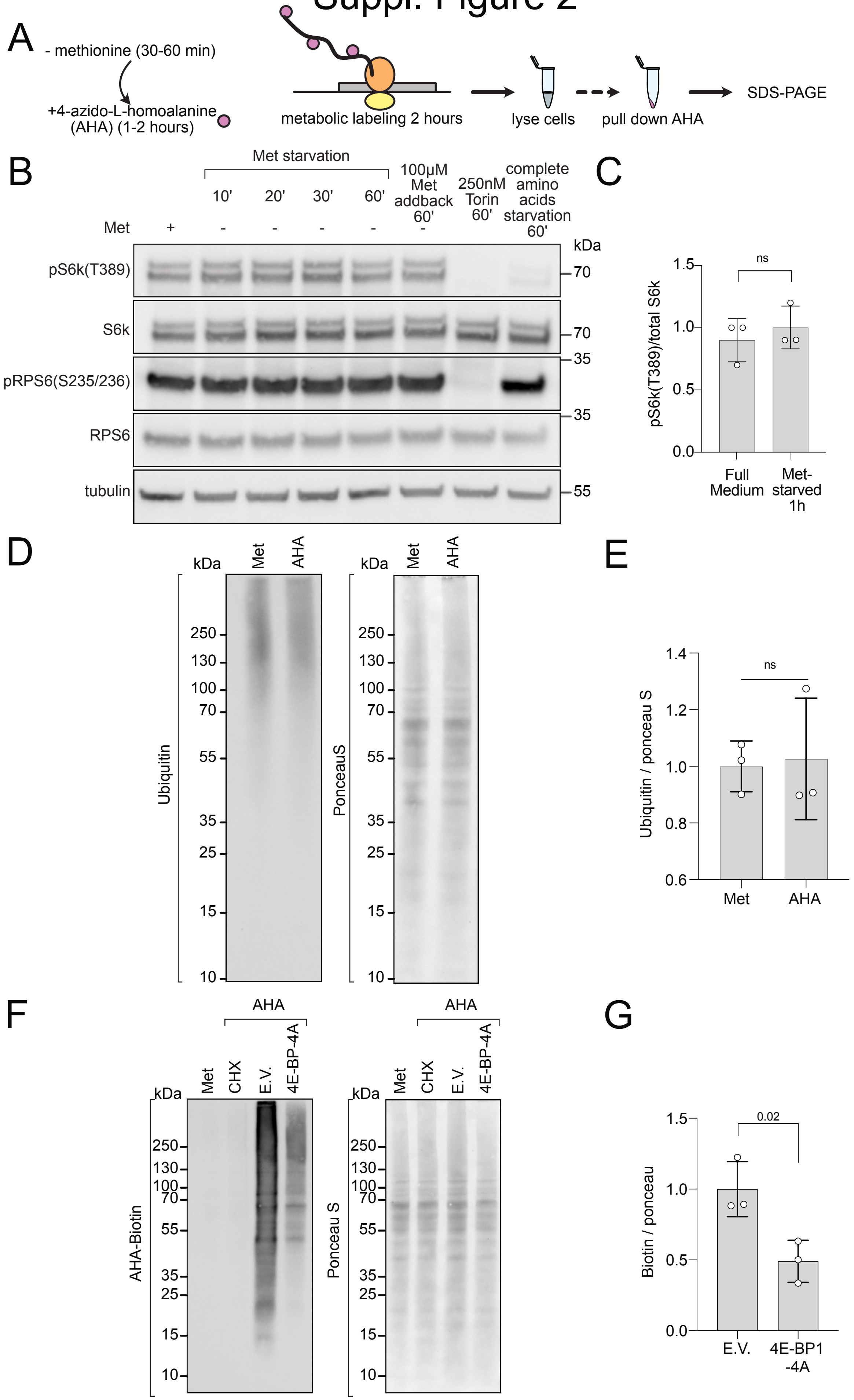

t

Suppl. Figure 2: Setup and control experiments for de novo translation BONCAT assay.

(A) Schematic diagram illustrating the BONCAT approach for assaying newly synthesized proteins. Newly synthesized proteins are labeled in vivo using a pulse of metabolic labeling whereby methionine is removed from the medium and replaced with 4-azido-L-homoalanine (AHA) for 2 hours. Cells are then lysed, and AHA is linked to biotin using click-chemistry. The biotin labeled proteins are then pulled down with neutravidin and subjected to SDS-PAGE.

(B-C) Starvation specifically for methionine does not lead to inhibition of mTORC1 within a 60-minute timeframe. Torin and complete amino acid starvation are positive controls. (B) Representative immunoblot. (C) Quantification of 3 biological replicates.

(D-E) Replacement of methionine for 4-azido-L-homoalanine (AHA) in the medium does not lead to changes in global protein degradation, assayed via levels of ubiquitination with pan-ubiquitin antibodies. (D) Representative immunoblot. (E) Quantification of 3 independent biological replicates.

(F-G) BONCAT detects the general drop in translation caused by 4E-BP1-4A expression. (F) Representative immunoblot where all AHA-biotin labeled protein are detected. (G) Quantification of three biological replicates. Error bars represent standard deviation. Significance by unpaired, two-sided, t-test. ns=not significant.

Suppl. Figure 3

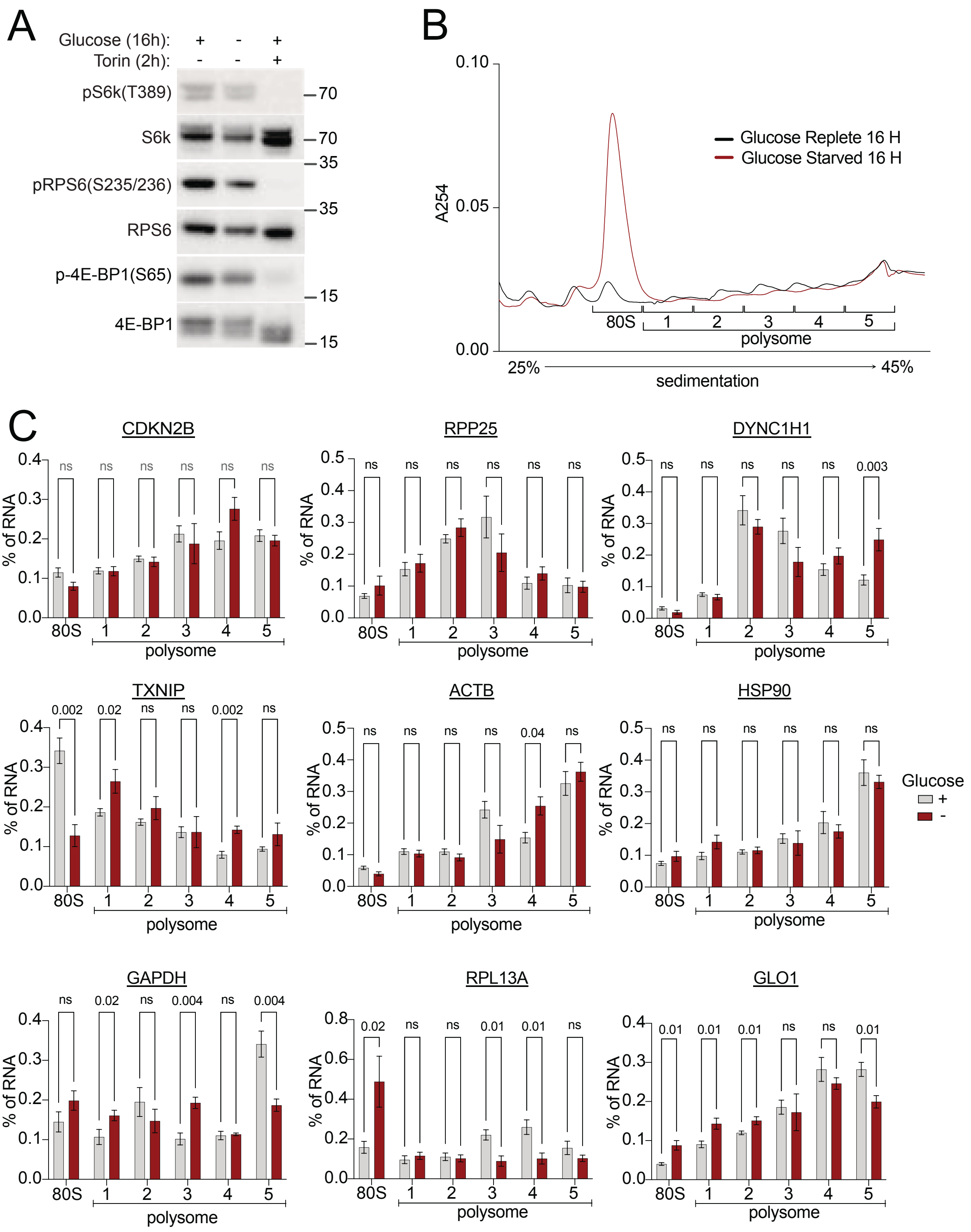

Suppl. Figure 3: Resistant mRNAs continue being translated when cells are starved of glucose

(A) Glucose removal causes partial inhibition of mTORC1, detected via phosphorylation of the direct target S6K, and the indirect target RpS6. Torin is used as a positive control.

(B) Glucose removal causes a reduction in the polysome peaks and an increase in the size of the monosome peak. The 80S fraction and the polysome fractions used for Q-RT-PCR in panel C are indicated.

(C) Upon glucose removal, resistant mRNAs such as *CDKN2B, RPP25* and *DYNC1H1* do not shift out of polysomes as much as control mRNAs such as *GAPDH, RPL13A* or *GLO1*. Endogenous mRNAs were detected by Q-RT-PCR from the various fractions, spiked with Renilla Luciferase mRNA as a normalization control. Error bars=std. dev., significance by unpaired, two-sided, t-test.

Suppl. Figure 4

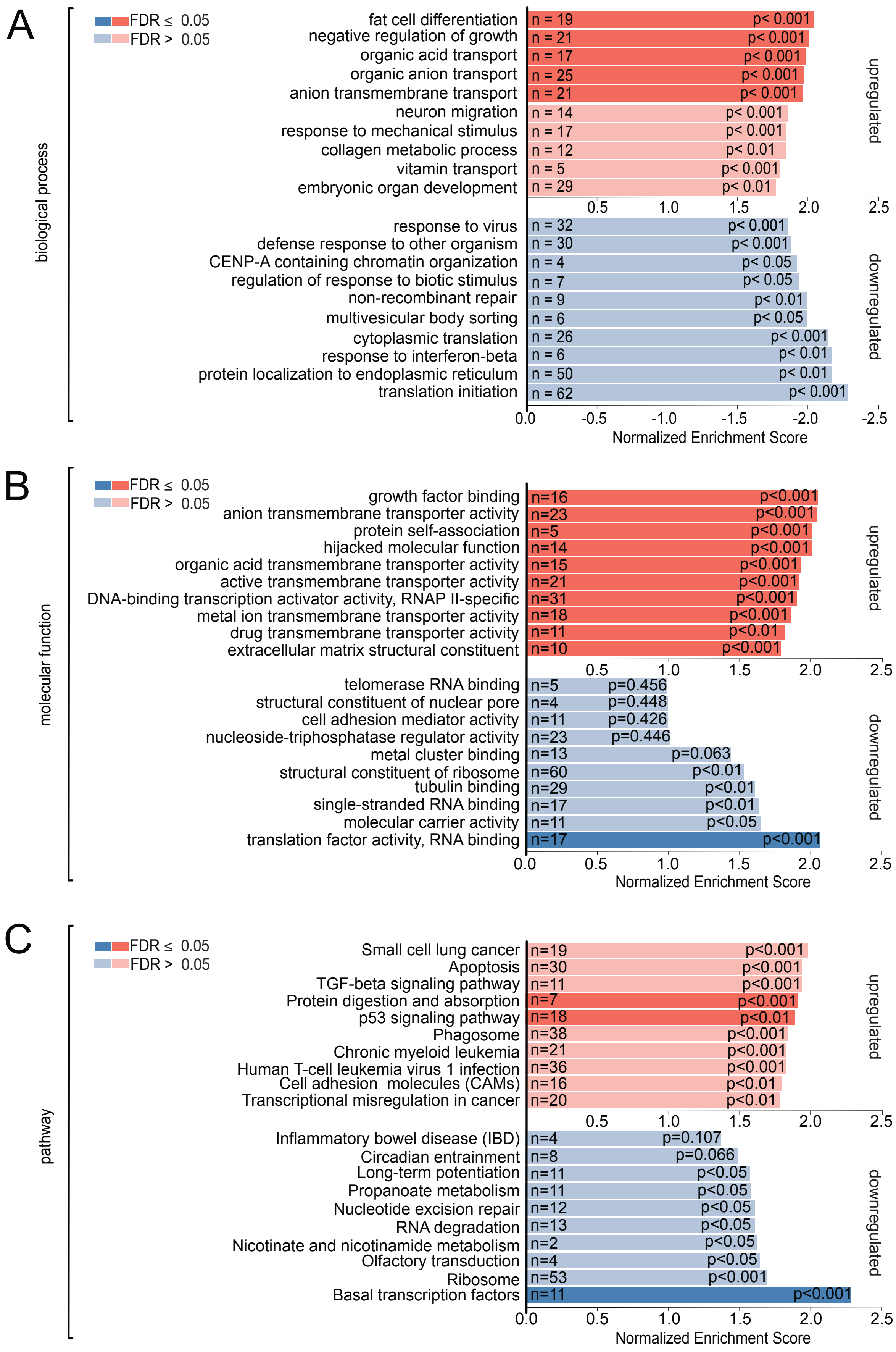

Suppl. Figure 4: Gene Set Enrichment Analysis (GSEA) for transcripts that are either resistant or sensitive to eIF4E inhibition.

Gene Set Enrichment Analysis was done on the entire ribosome footprinting dataset, sorted by log2(fold change) in translation efficiency upon 4E-BP-4A expression. The results for the following categories are depicted: biological process (A), molecular function (B) and pathway (C). The terms with false discovery rates (FDR) less than 0.5 are presented as dark blue and dark red, with FDR more than 0.5 as light blue and light red. The p-values and number of genes for each group are indicated.

Suppl. Figure 5

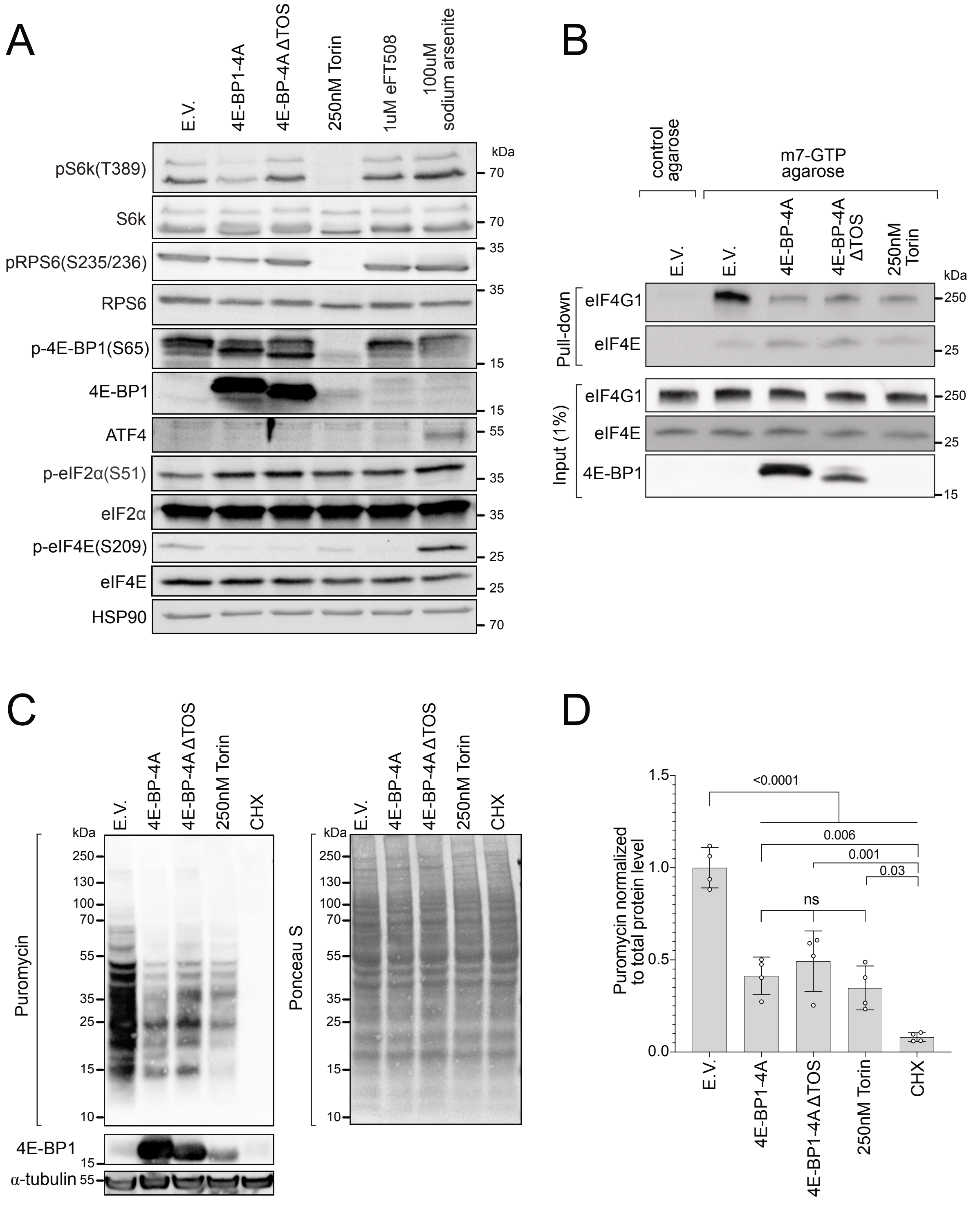

Suppl. Figure 5: 4E-BP1-4AΔTOS still inhibits eIF4G binding to eIF4E without perturbing mTORC1 activity.

(A) Unlike 4E-BP1-4A, expression of 4E-BP1-4A-ΔTOS does not reduce mTORC1 activity, detected via phosphorylation of the direct target S6K. Torin is used as a control for mTORC1 inhibition, the MNK1/2 inhibitor eFT508 as a control for eIF4E dephosphorylation and sodium arsenite as a control for activation of the integrated stress response and ATF4 induction.

(B) 4E-BP1-4A-ΔTOS blocks binding of eIF4G to eIF4E. Lysates from HeLa cells overexpressing either 4E-BP1-4A, 4E-BP14AΔTOS, or empty vector (E.V.) were subjected to pull-down on m-7-GTP sepharose beads. Full-length and ΔTOS 4E-BP1-4A disrupt eIF4G1 binding to eIF4E. The effect is comparable with 1 hour 250 nM Torin treatment.

(C-D) 4E-BP1-4A-ΔTOS blocks global translation as well as 4E-BP1-4A or Torin, detected via incorporation of OPP. Cells overexpressing either 4E-BP1-4A, 4E-BP1-4AΔTOS, or empty vector were treated with OPP for 1 hour. Treatment with 250nM Torin was used as control for inhibition of mTORC1 activity. Changes in global protein translation levels were assessed by western blot against OPP and normalized to total protein amount assayed by PonceauS. (C) Representative blots. (D) Quantification of three independent replicates. Error bars represent standard deviation. Significance by Tukey's multiple comparison test ANOVA. ns=not significant.

Suppl. Figure 6

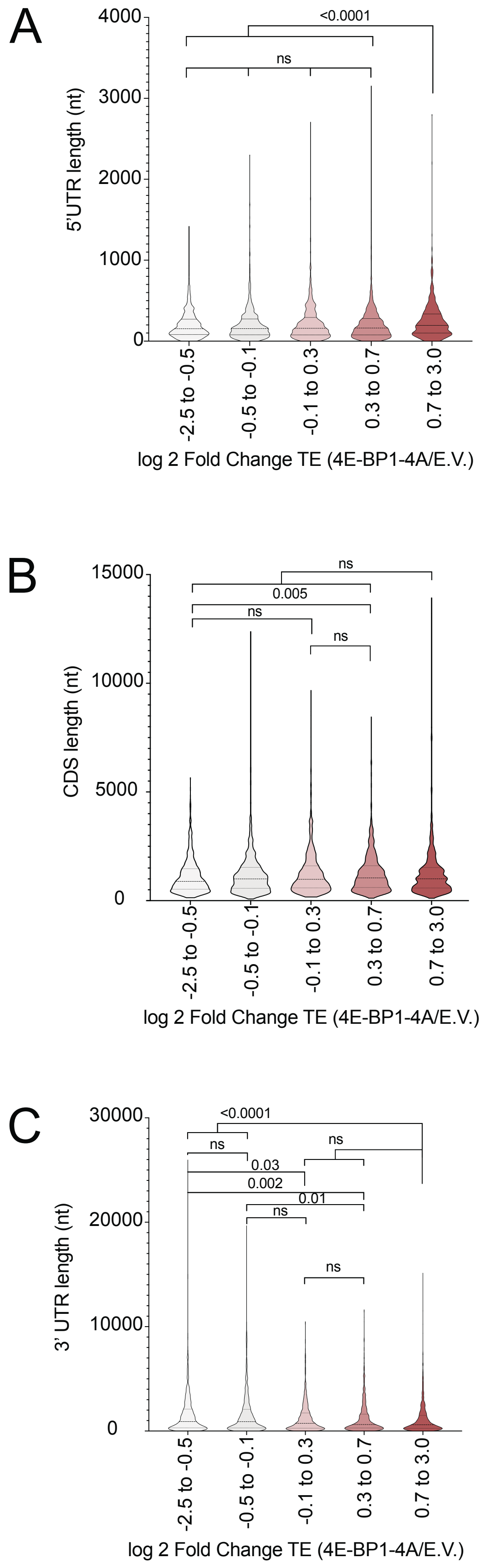

Suppl. Figure 6: Correlation of ribosome profiling results upon 4E-BP-4A expression versus different mRNA parameters.

All mRNAs detected in the footprinting experiment from HeLa cells were placed into buckets according to the log2(fold change) in translation efficiency caused by 4E-BP1-4A expression. The distribution is then plotted for each bucket for the following mRNA parameters: 5' UTR length (A), CDS length (B), or 3'UTR length (C). Significance by two-sided Kruksal Wallis tests, adjusted for multiple testing. ns=not significant.

Suppl. Figure 7

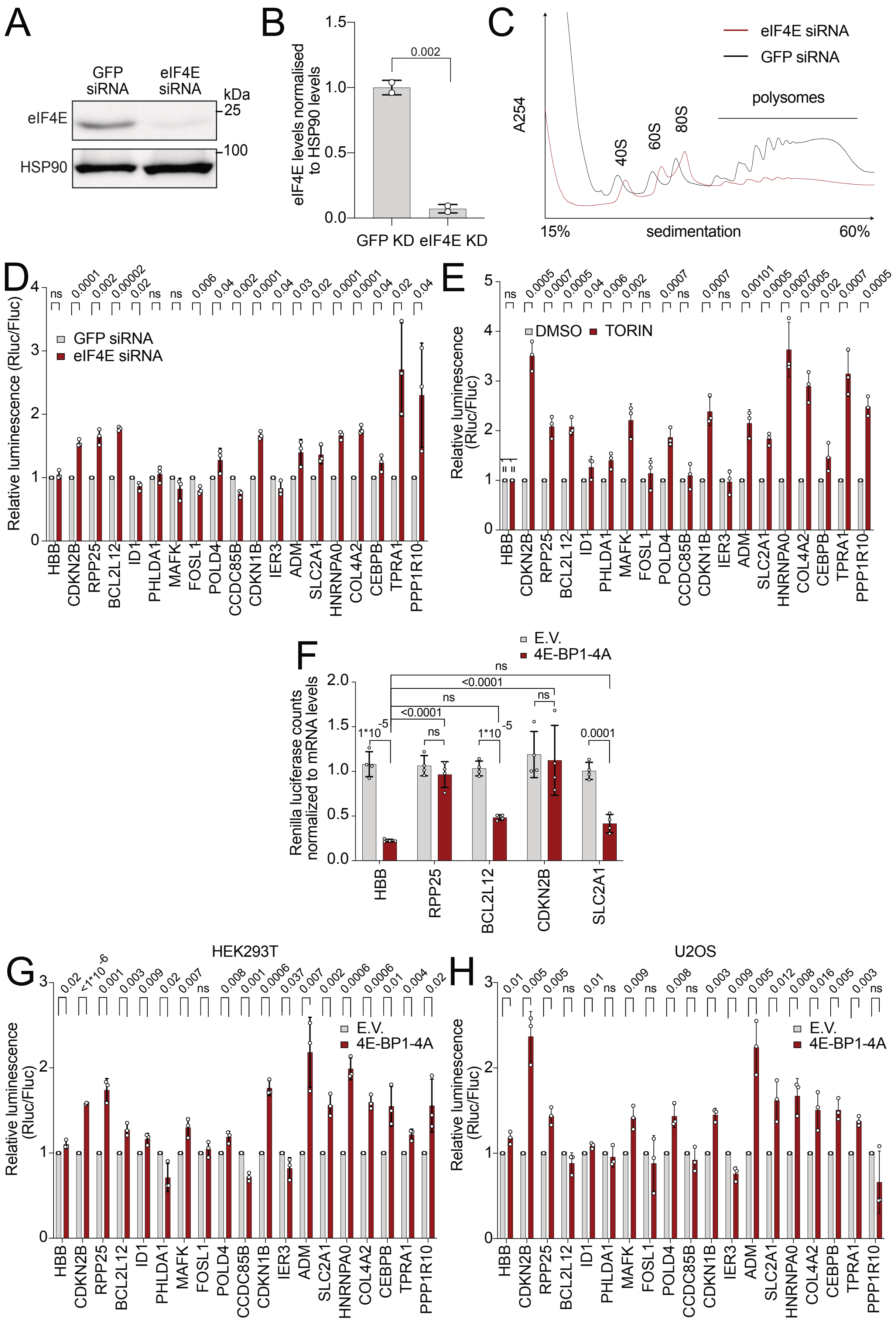

Suppl. Figure 7: Support to Main Figure 2

(A-B) Validation of *eIF4E* knock-down by immunoblotting. (A) Representative immunoblot blot of lysates of HeLa cells transfected either with siRNA targeting *eIF4E*, or *GFP* as a negative control. (B) Quantification of knockdown efficiency from three independent replicates.

(C) *eIF4E* knock-down reduces but does not completely blunt global translation, assessed via polysome profile.

(D-E) Luciferase reporters carrying the 5'UTRs of 'resistant' transcripts show translation levels that are higher than the *HBB* control reporter when eIF4E is inhibited either with an siRNA targeting *eIF4E* (negative control=siRNA targeting *GFP*) (D) or with Torin (E). The same reporters as in main Fig. 2A were treated either with DMSO or 250nM Torin for 16 hours or transfected either in cells with *GFP* or *eIF4E* knock-down. Three independent biological replicates were quantified. Error bars represent standard deviation. Significance by unpaired, two-sided, t-test adjusted for multiple testing. ns=not significant.

(F) Confirmation that luciferase reporters carrying the 5'UTRs of 'resistant' transcripts show elevated expression upon eIF4E inhibition due to a translational effect, quantified as raw renilla luciferase counts normalized to reporter mRNA levels quantified by qRT-PCR. Four independent biological replicates were quantified. Error bars represent standard deviation. Significance by unpaired, two-sided, t-test adjusted for multiple testing. ns=not significant.

(G-H) Luciferase reporters carrying the 5'UTRs of transcripts that are resistant to eIF4E inhibition in HeLa cells are also resistant to eIF4E inhibition in

HEK293T or U2OS cells. Luciferase reporters carrying the indicated 5’ UTRs were transfected into either HEK293T (G) or U2OS (H) cells, together with 4E-BP1-4A to inhibit eIF4E, or with an empty vector (E.V.) as a negative control. Three independent biological replicates were quantified. Error bars represent standard deviation. Significance by unpaired, two-sided, t-test adjusted for multiple testing. ns=not significant.

Suppl. Figure 8

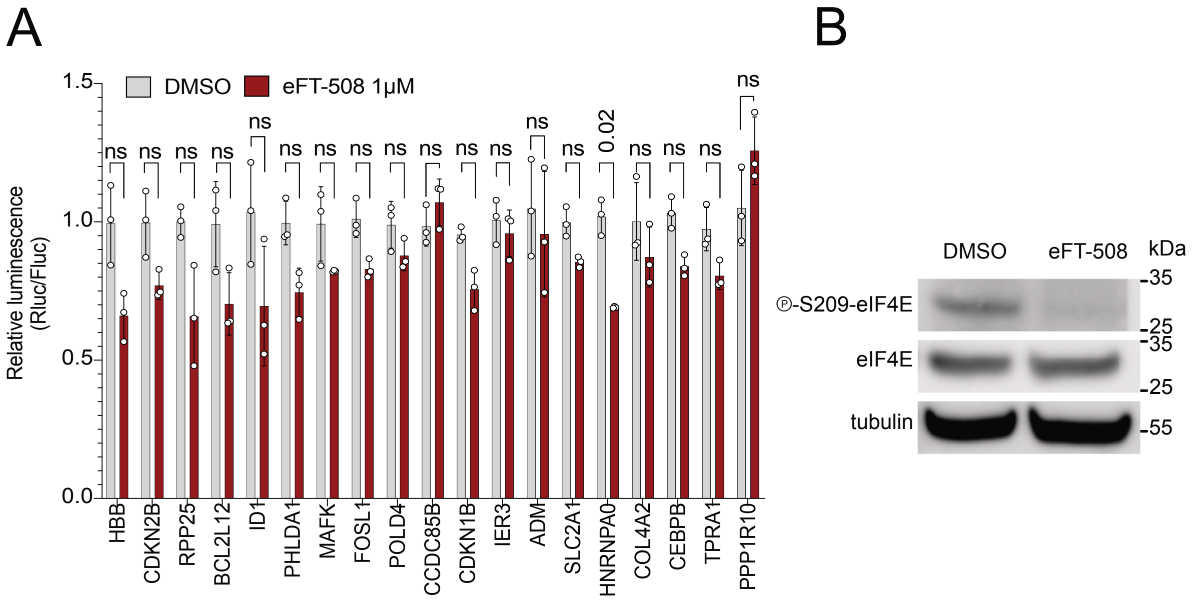

Suppl. Figure 8: Dephosphorylation of eIF4E does not stimulate translation of resistant reporters.

(A) Unlike eIF4E inhibition via 4E-BP-4A expression, Torin, or *eIF4E* knockdown, inhibition of eIF4E phosphorylation with eFT-508 does not stimulate translation of resistant reporters in comparison to the untreated condition. n = 3 biological replicates. Error bars represent standard deviation. Unpaired, two-sided, t-test adjusted for multiple testing: ns= not significant.

(B) Immunoblot validation that eFT-508 treatment (1μM, 2 hours) causes dephosphorylation of eIF4E.

Suppl. Figure 9

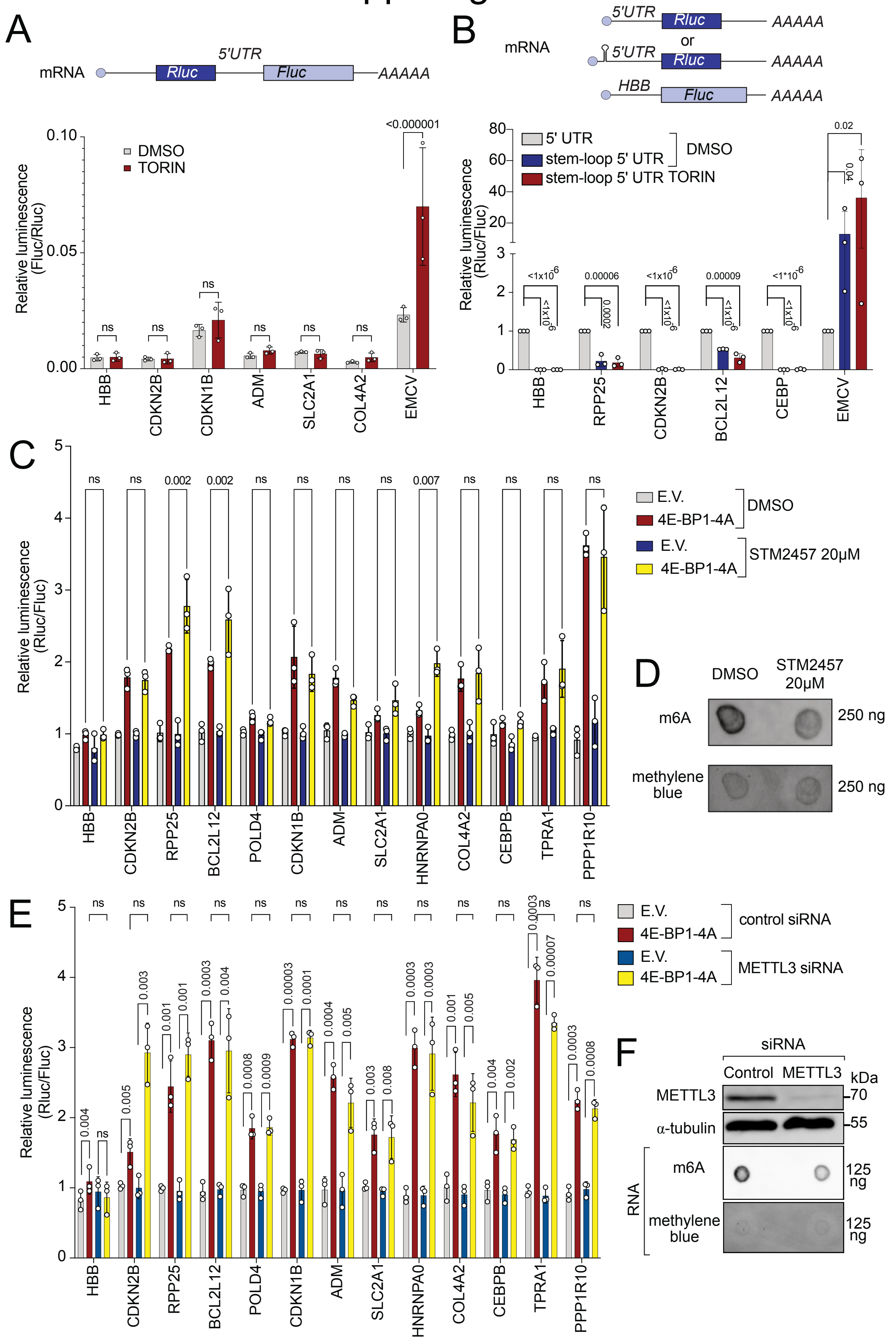

Suppl. Figure 9: Resistant transcripts rely on 5'-end dependent translation initiation upon eIF4E inhibition.

(A) The 5'UTRs of resistant mRNAs do not show increased activity upon eIF4E inhibition with Torin, when not placed at the 5'end of a transcript. Bicistronic reporters carrying the indicated 5'UTR were transcribed in vitro and transfected as mRNA into cells co-treated with +/- 250nM Torin for 6 hours. Only the EMCV IRES element showed an increase in translation upon co-treatment with Torin. Three independent biological replicates were quantified.

(B) Introduction of a stem-loop at the 5'end of resistant reporters causes a dramatic reduction in expression, indicating that their translation is normally 5'-end dependent, and this residual translation does not increase upon eIF4E inhibition with Torin (250 nM, 6 hours). The EMCV IRES is used as a positive control. n = 3 biological replicates x 4-8 technical replicates.

(C-F) Resistant reporters are still resistant to eIF4E inhibition when RNA methylation on m6A is inhibited either pharmacologically (C-D) or with a *METTL3* knockdown (E-F). (C) Pharmacological inhibition of METTL3 by 16h treatment with 20μM STM2457 does not affect resistance of the reporters to eIF4E inhibition (compare yellow to red bars). (D) Dot blot validation to determine the m6A levels after 16h of STM2457 treatment. (E) Knockdown of *METTL3* does not affect resistance of the reporters to eIF4E inhibition (compare yellow to red bars). (F) *METTL3* knockdown efficiency assessed by western blot (top panel) and m6A-dot blot of total RNA (bottom panel)

All panels: Error bars represent standard deviation, significance by unpaired, two-sided, t-test adjusted for multiple testing (A) or by Dunnett's multiple comparison test ANOVA (B,C,E). ns=not significant.

Suppl. Figure 10

A

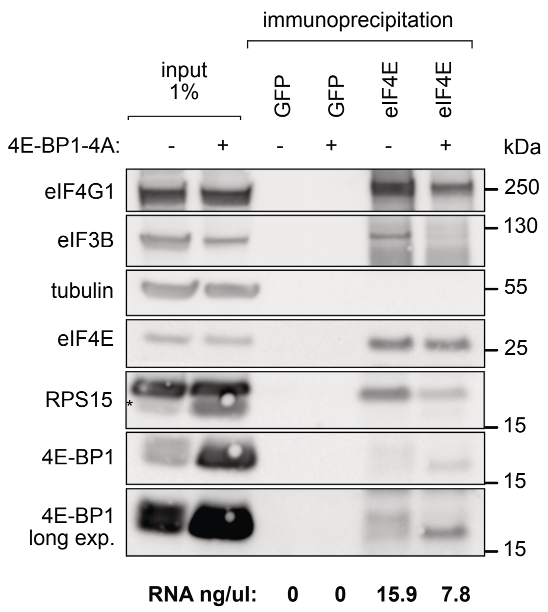

Suppl. Figure 10: Control blot for eIF4E immunoprecipitation and levels of RNA co-IPing in the various samples.

(A) Western blot control for eIF4E-immunoprecipitation. The negative control antibody, anti-GFP, does not IP protein or RNA. Instead, eIF4E-antibodies IP eIF4E and other proteins associated with eIF4E such as eIF4G1 or ribosomal proteins, as well as RNA. eIF4E IP from cells overexpressing 4E-BP1-4A shows a reduction in the co-IPed eIF4G1, eIF3b and RPS15, while the IPed 4E-BP1 signal increases. *the remaining signal from 4E-BP1 antibodies, because the 4E-BP1 blot was re-blotted to detect RPS15.

Suppl. Figure 11

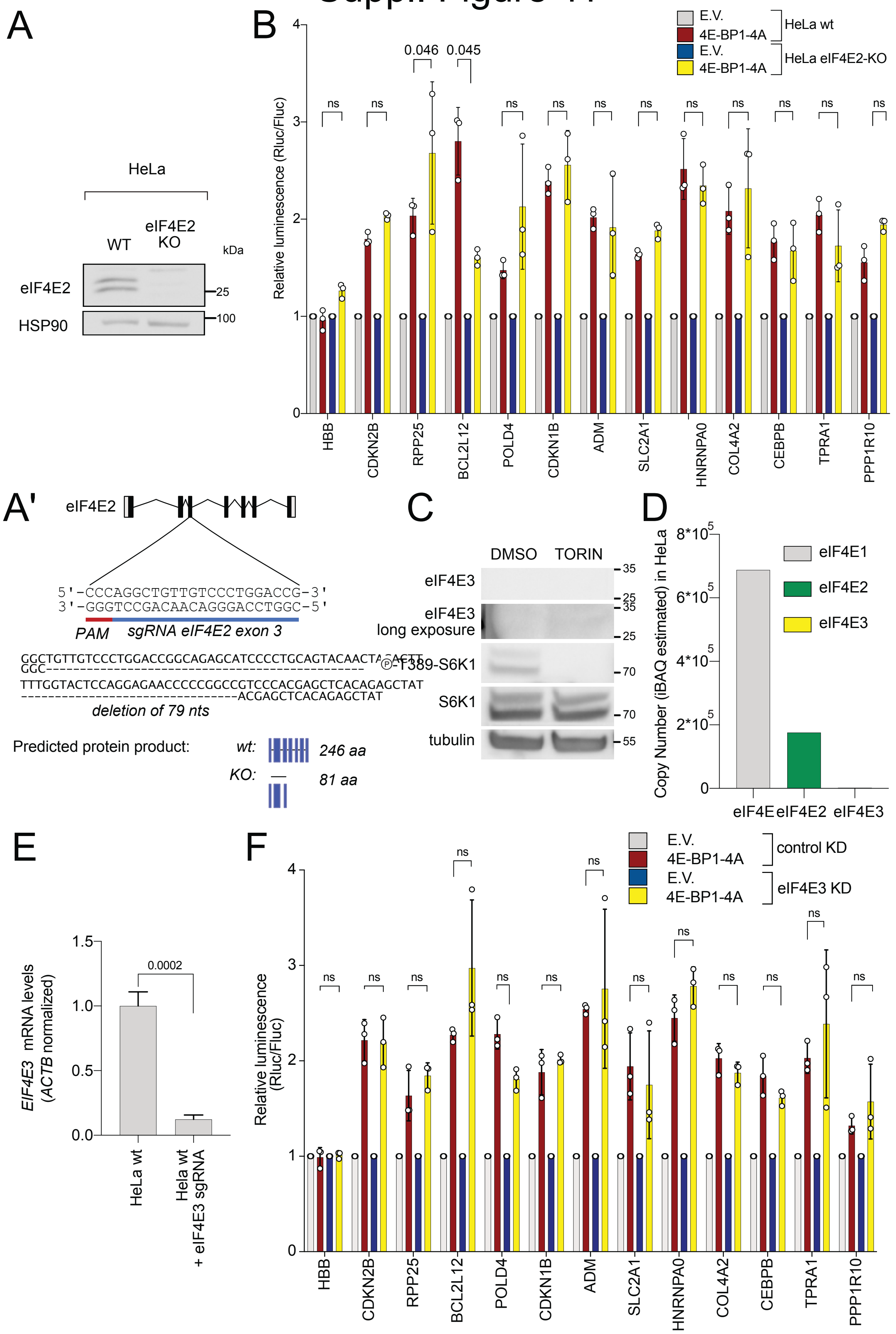

Suppl. Figure 11: eIF4E2 and eIF4E3 do not contribute to the eIF4E1-independent translation of resistant reporters.

(A-A') Loss of eIF4E2 in the *eIF4E2* knock-out cell line, validated by western blot (A) and genotyping (A'). Editing events in the eIF4E2-KO line at the genomic levels are shown in (A')

(B) Loss of eIF4E2 does not affect the resistance of reporters to eIF4E1 inhibition, except *BCL2L12*. Significance by Dunnett's multiple comparison test ANOVA. ns= not significant.

(C) Antibodies against eIF4E3 do not detect any eIF4E3 protein in HeLa cells.

(D) Quantification of expression levels of the three eIF4E isoforms in Hela cells by mass spectrometry from [1].

(E) Knockdown efficiency of *eIF4E3* by qRT-PCR. Significance by unpaired, two-sided, t-test adjusted for multiple testing

(F) *eIF4E3* knock-down does not affect the resistance of reporters. Compare red to yellow bars. Significance by Dunnett's multiple comparison test ANOVA. ns= not significant.

Suppl. Figure 12

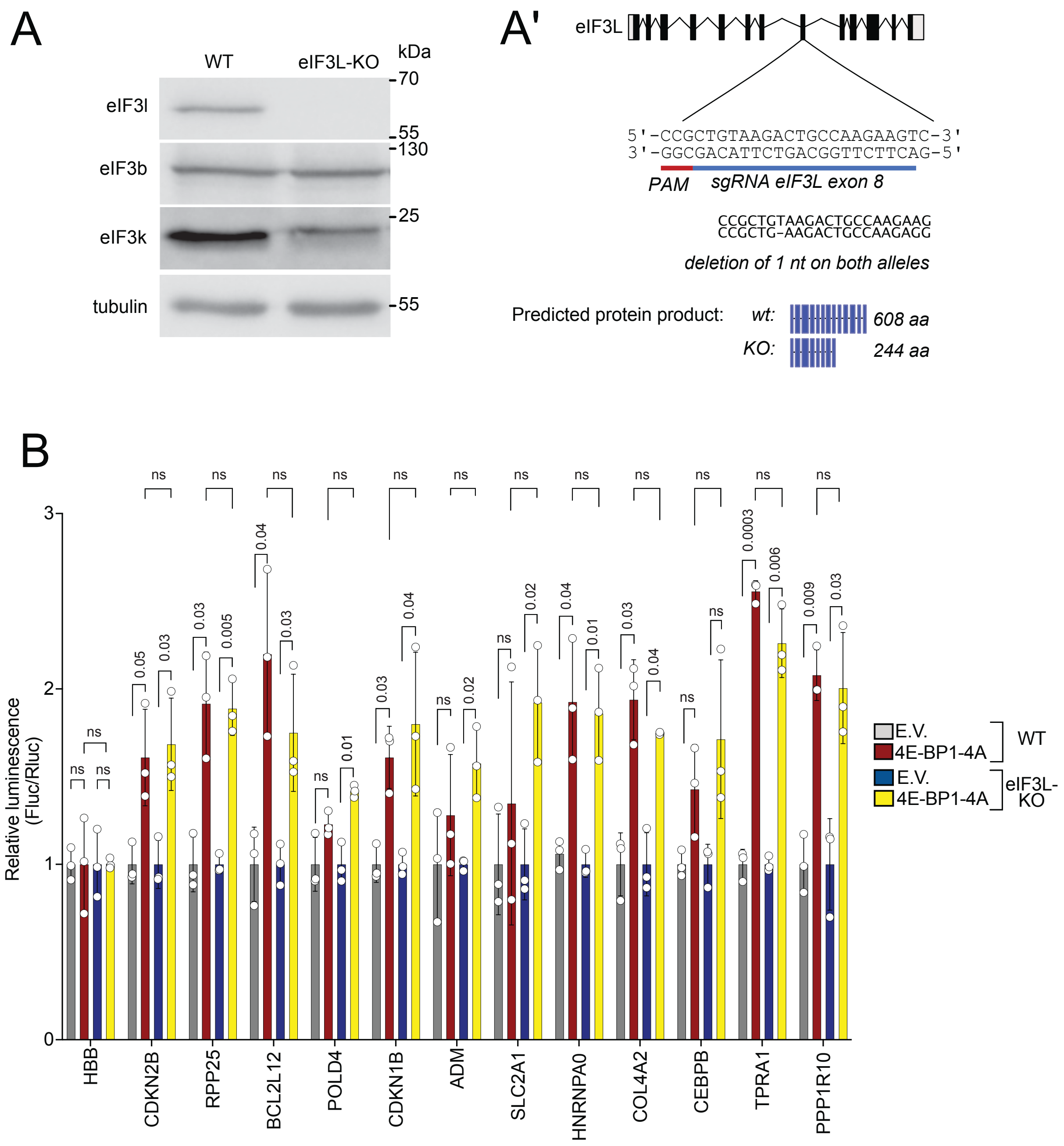

Suppl. Figure 12: eIF3l does not contribute to translation of the resistant reporters upon eIF4E inhibition.

(A) Loss of eIF3l in the *eIF3l*-KO HeLa cell line, validated by immunoblotting. A decrease in levels of the eIF3l-interacting partner eIF3k is detected.

(A') Molecular characterization of the *eIF3l* alleles in the *eIF3l*-KO cell line.

(B) *eIF3l* knock-out does not affect the resistance of reporters to inhibition of eIF4E. Significance by Dunnett's multiple comparison test ANOVA. ns= not significant.

Suppl. Figure 13

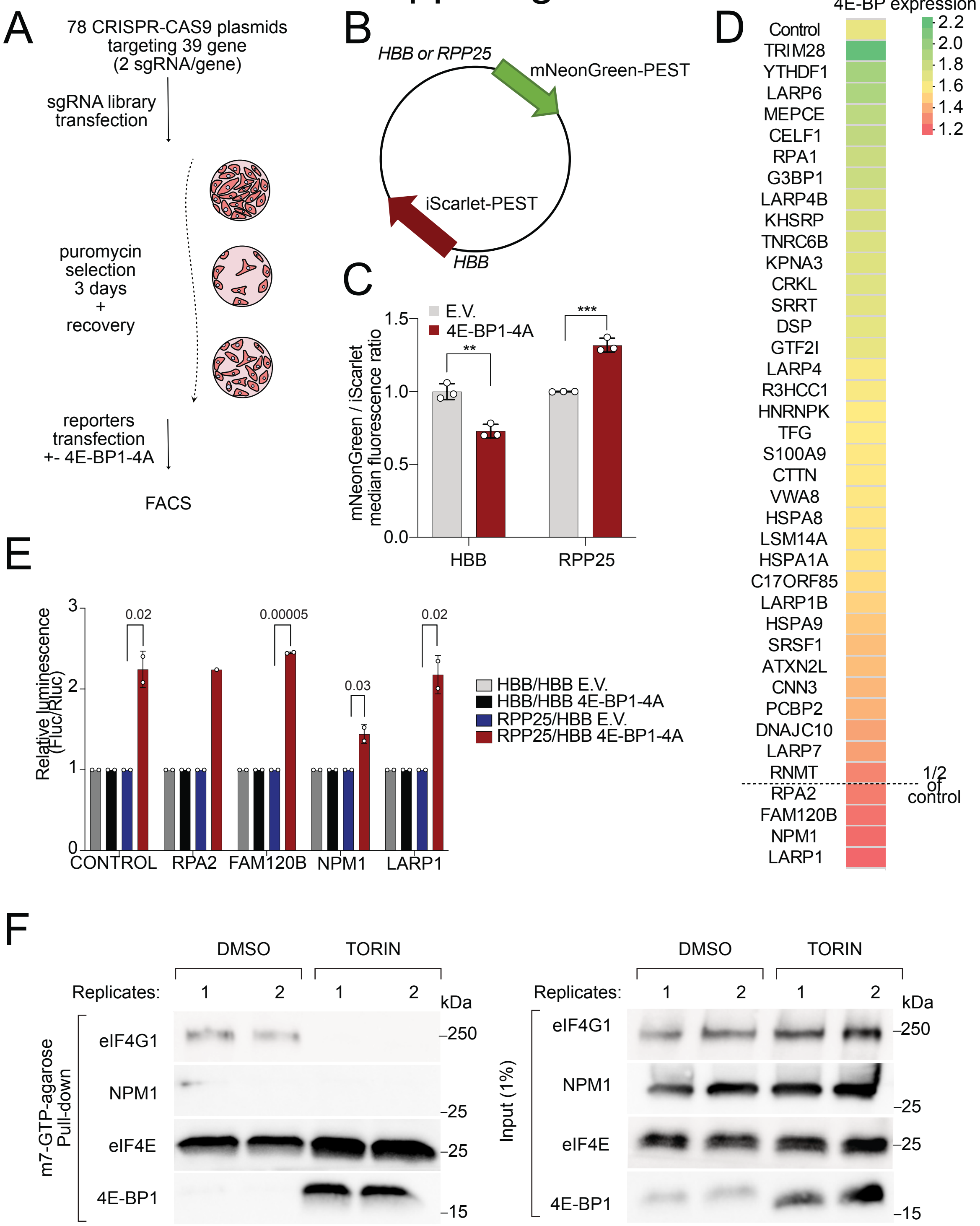

Suppl. Figure 13: Targeted screen on cap-binding proteins for proteins required for resistance to eIF4E inhibition.

(A) Schematic illustration of the screen set-up. 39 genes encoding cap-binding proteins were targeted by CRISPR/Cas9, each with 2 sgRNA pooled together. After transfection with sgRNAs and selection for 3 days, the pool of cells was allowed to grow for a couple days before transfecting with reporters and performing luciferase assays.

(B) Schematic illustration of the fluorescent reporter used for screening. A PEST domain was introduced to the C-terminus of NeonGreen and iScarlet to reduce their half-lives. The mNeonGreen ORF was preceded either by the HBB negative control 5'UTR, or by the 5'UTR of the resistant transcript RPP25.

(C) The fluorescent reporter recapitulates the resistance of *RPP25* translation to 4E-BP1-4A expression, as seen with luciferase reporters. Significance by unpaired, two-sided, t-test adjusted for multiple testing. ns= not significant.

(D) Heat map summarizing results of the targeted screen knocking out selected cap-binding proteins on resistance of the *RPP25* fluorescent reporter to eIF4E inhibition upon 4E-BP-4A expression. Half of the *RPP25* induction in HeLa wt cells is depicted with a dotted line.

(E) *NPM1* loss-of-function blunts the resistance of the RPP25 reporter to eIF4E inhibition. The top candidates from the fluorescent-based screen shown in Suppl. Fig. 13 are retested here using luciferase reporters. Significance by unpaired, two-sided, t-test adjusted for multiple testing. ns= not significant.

(F) Little or no binding of NPM1 to cap. Lysates from HeLa cells treated with DMSO or Torin were subjected to pull-down on m-7-GTP sepharose beads. Two replicates are shown. No reproducible NPM1 was detected on cap-beads in either condition.

Suppl. Figure 14

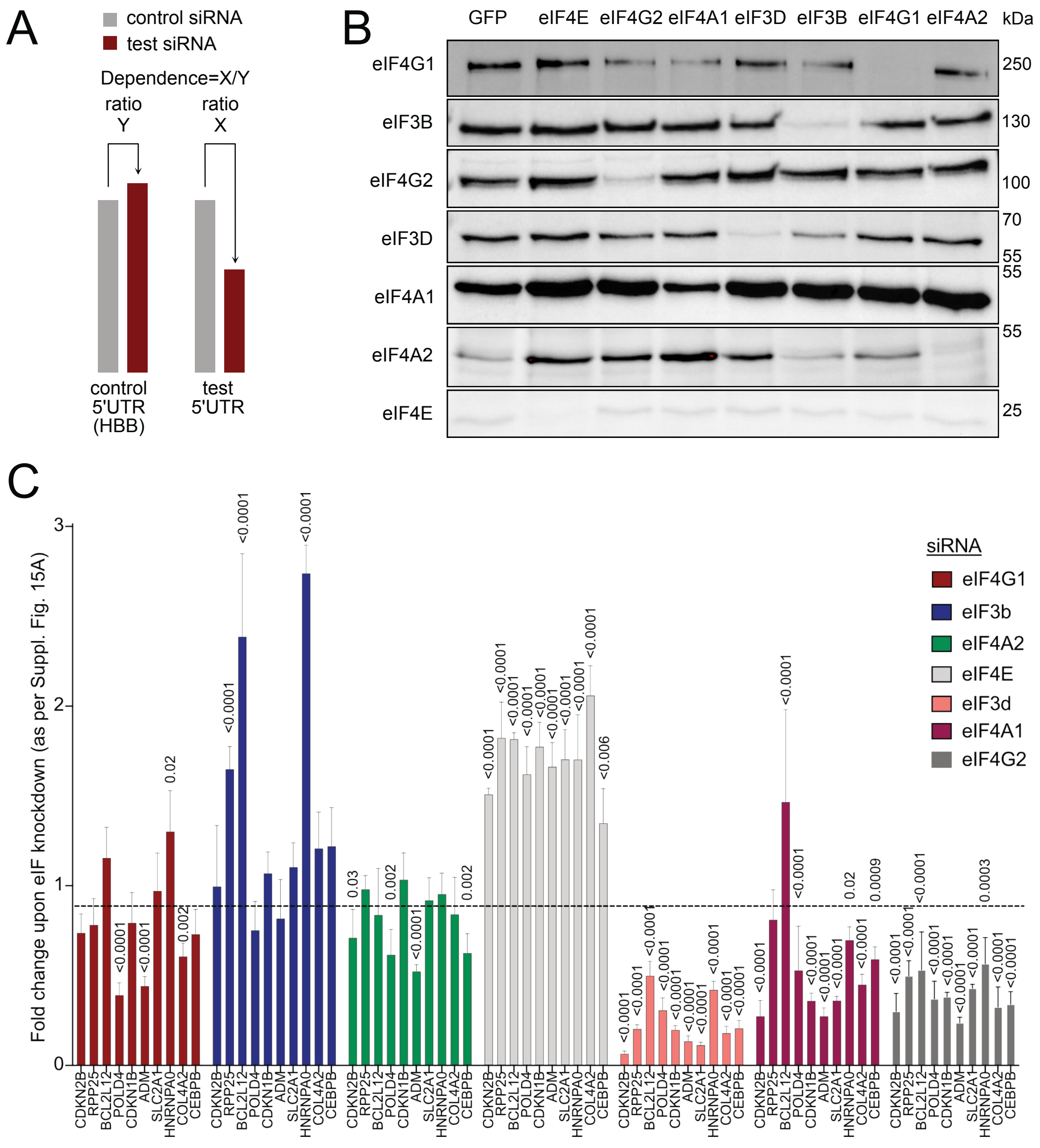

Suppl. Figure 14: Translation of resistant reporters is highly dependent on eIF3d.

(A) Schematic illustration of how dependence to a gene knockdown is quantified. First, all RLuc signals are normalized to the co-transfected FLuc normalization control (not shown). Next, for each reporter, the values in the presence of the gene knockdown were divided by the values in the absence of the gene knockdown. Finally, for each Reporter of interest (ROI), this value was normalized by the value of the *HBB* reporter, essentially setting the negative control *HBB* reporter to 1.

(B) Western blot validation for knock-down efficiency of various eIFs.

(C) Translation of resistant reporters is highly dependent on eIF3d. The indicated reporters were transfected into cells that had been treated with siRNAs targeting the indicated *eIF* genes. Dependence is quantified as shown in panel A. Significance by Dunnett's multiple comparison test ANOVA. ns=not significant.

Suppl. Figure 15

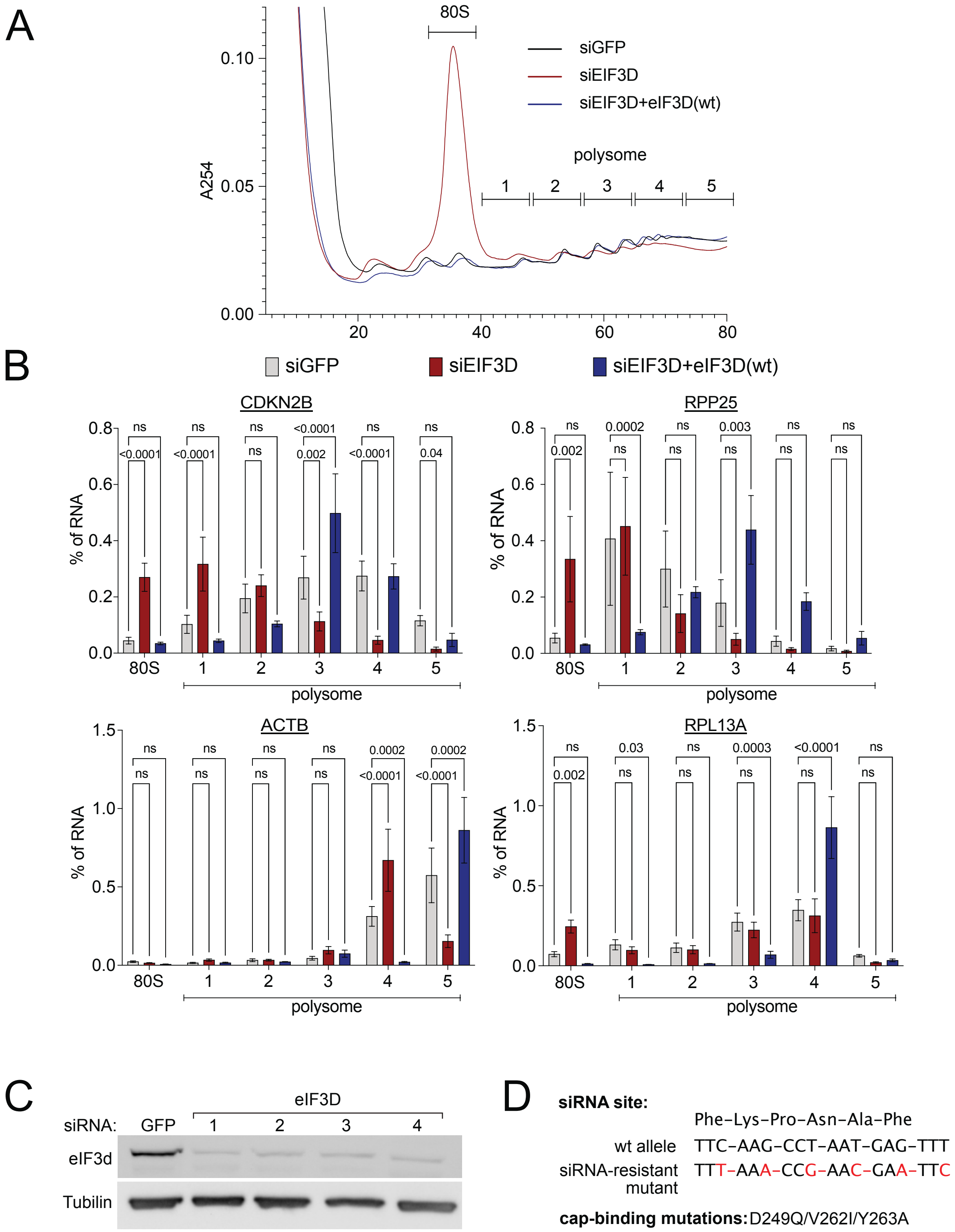

Suppl. Figure 15: Resistant transcripts shift out of polysomes upon *eIF3d* knockdown in non-stressed conditions.

(A) Polysome profiles of HeLa cells with either control knock-down, *eIF3d*-knokdown or *eIF3d* knock-down reconstituted to express a variant of eIF3d containing silent mutations that make it resistant to the siRNA.

(B) Transcripts resistant to eIF4Einhibition, such as *RPP25* or *CDKN2B*, shift more strongly out of polysomes upon *eIF3d* knockdown than other control transcripts, *ACTB* and *RPL13a*. Distribution of endogenous transcripts within the polysome profiles is shown, detected by Q-RT-PCR from the fractions shown in panel C, normalized to an exogenous Renilla luciferase mRNAs that was spiked into the fractions as a normalization control. Significance by Dunnett's multiple comparison test ANOVA. ns=not significant.

(C) Identification of a single siRNA with good knockdown efficiency. Four siRNAs were tested for knock-down efficiency by immunoblotting. siRNA #1 was selected.

(D) The seed region targeted by *eIF3d*-siRNA #1 was mutated in the eIF3d coding sequence using synonymous mutations, as shown. Additionally, the mutations published to affect eIF3d-cap binding are shown.

Suppl. Figure 16

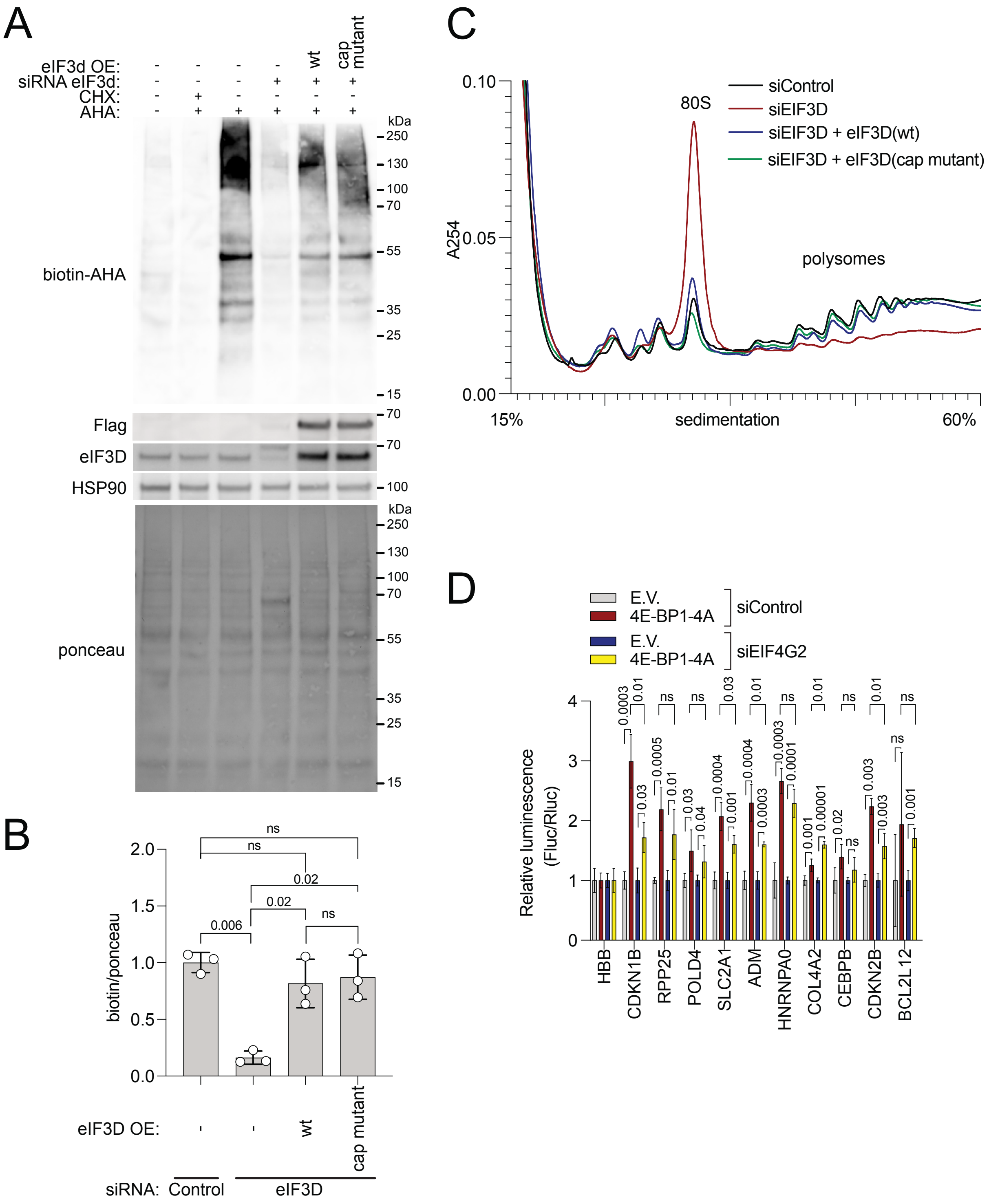

Suppl. Figure 16: Generation of HeLa cell lines expressing eIF3d versions resistant to the *eIF3d* siRNA.

(A-C) Reconstitution of *eIF3d* knockdown cells with either wildtype eIF3d or an eIF3d mutant lacking cap binding restores bulk translation rates to near-normal, assayed via incorporation of AHA (A-B) or by polysome profiles (C). Thus either variant of eIF3d can rescue the translation inhibition caused by *eIF3d* knock-down. (A) Representative blots. (B) Quantification of 3 biological replicates. Significance by Dunnett's multiple comparison test ANOVA. ns= not significant. (C) Polysome profiles that corroborates the results observed by AHA-assay. Strong accumulation of an 80S peak is only observed in HeLa cells with *eIF3d* siRNA knock-down.

(D) *eIF4G2* knockdown blunts the resistance of reporters to eIF4E inhibition. Reporters were transfected into control or *eIF4G2* knock-down cells, and co-transfected with 4E-BP-4A to inhibit eIF4E or with an empty vector (E.V.). Significance by unpaired, two-sided, t-test adjusted for multiple testing. ns= not significant.

Suppl. Figure 17

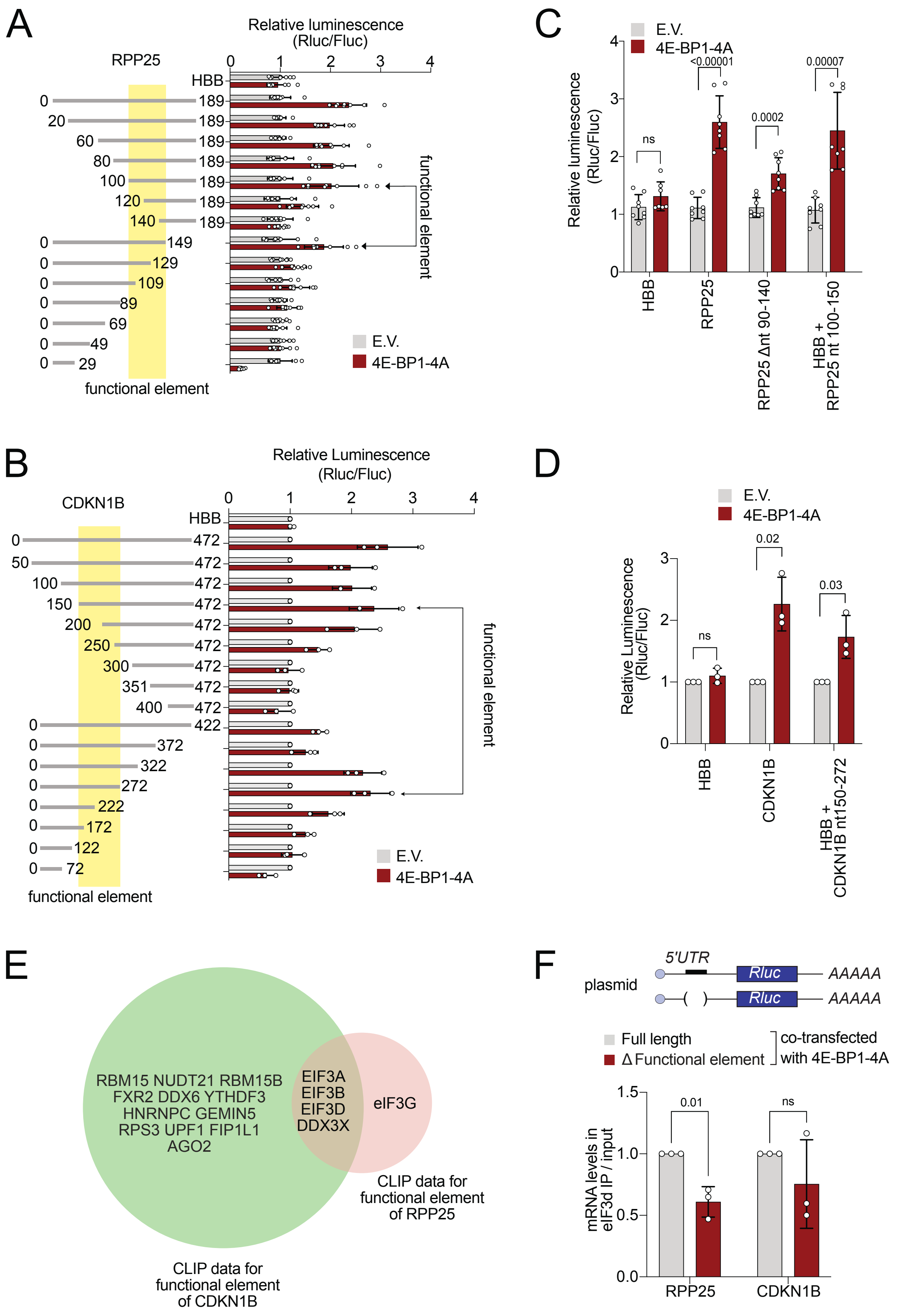

Suppl. Figure 17: Functional elements of the *RPP25* and *CDKN1B* mRNAs are identified as eIF3d-binding sites.

(A-B) Serial truncations of the *RPP25* (A) and *CDKN1B* (B) 5'UTRs in the luciferase reporters identifies the regions required for resistance to eIF4E inhibition. Truncations were performed from the 5' end and the 3' end. Deletion of the region shown in yellow leads to loss of resistance.

(C) A 50nt region of the *RPP25* 5'UTR is both required and sufficient to render the reporter resistant to eIF4E inhibition. Removal of the 50nt functional element (nt 90-140) from the *RPP25* 5'UTR abrogates resistance of the reporter, while placement of this functional element into the negative control *HBB* 5'UTR causes it to become resistant to eIF4E inhibition.

(D) Placement of a 122nt region from the *CDKN1B* 5'UTR into the negative control *HBB* 5'UTR causes it to become resistant to eIF4E inhibition.

(E) List of proteins identified by CLIP to interact with the functional elements of *RPP25* and *CDKN1B* identified in panels A-D from the POSTAR3 database [2].

(F) The *RPP25* and *CDKN1B* functional elements identified in panels A-B mediate binding to eIF3d. FLAG-eIF3d(WT) was immunoprecipitated from cells co-transfected with 4E-BP1-4A and RLuc reporters carrying either full-length wildtype *RPP25* or *CDKN1B* 5'UTRs or UTRs lacking the functional elements (Δ functional element). Co-IPed and input RNA were DNase treated, converted into DNA and subjected to qRT-PCR. IPed signal normalized to input is shown. Significance by unpaired, two-sided, t-test adjusted for multiple testing. ns= not significant.

Suppl. Figure 18

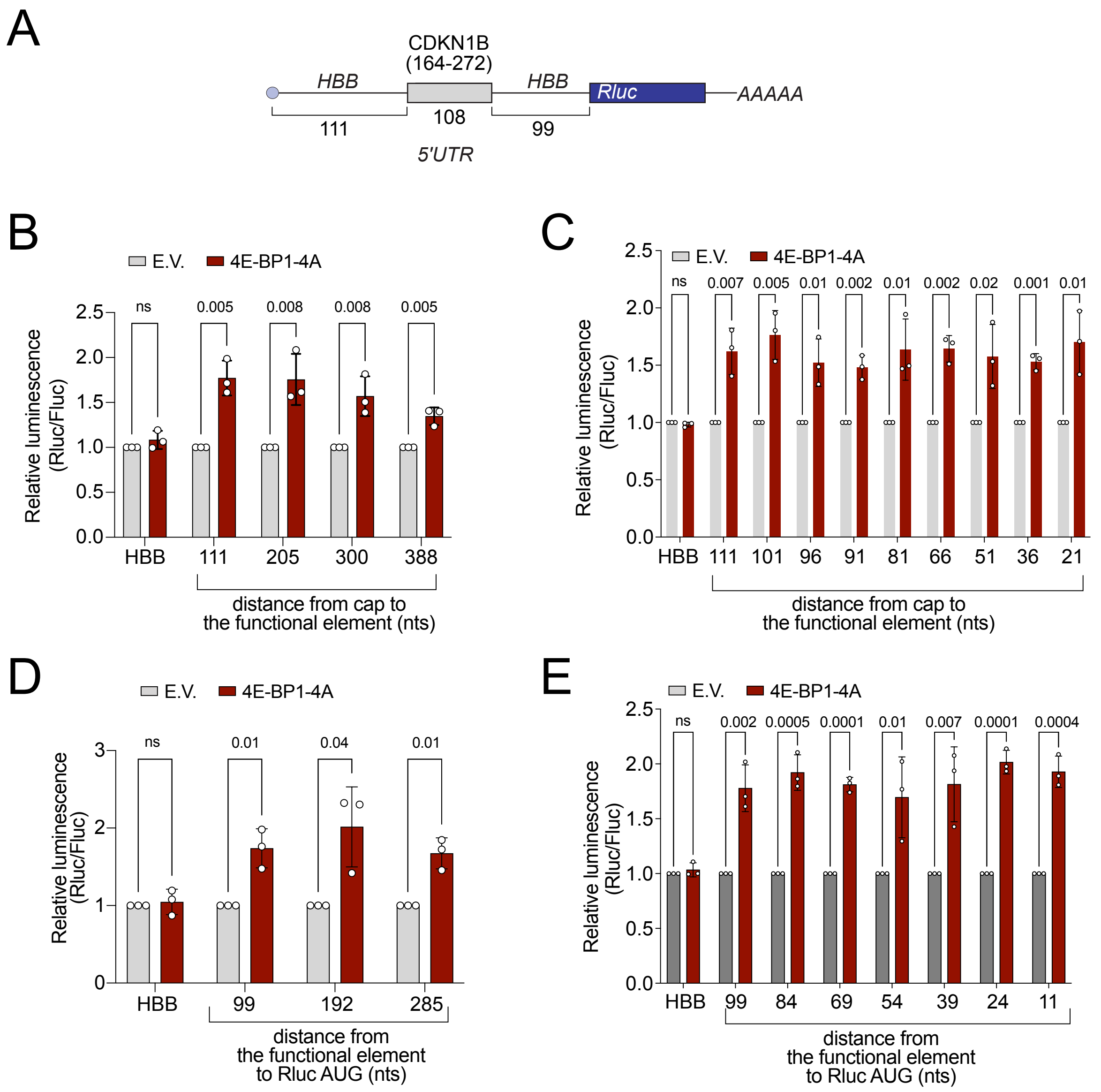

Suppl. Figure 18: The *CDKN1B* functional element loses activity if placed too far from the cap.

(A) Schematic representation of the RLuc reporter carrying the *HBB* 5'UTR into which the functional element of *CDKN1B* was inserted.

(B-C) Placement of the functional element further from the cap reduces its activity. Indicated reporters were transfected into HeLa cells overexpressing either empty vector or 4E-BP1-4A. Distance of the functional element to the cap was either increased via multimerization of the intervening sequence (B), or decreased via truncations (C).

(D-E) Distance of the *CDKN1B* functional element to the RLuc start codon does not affects its activity. Distance to the RLuc start codon was either increased via multimerization of the intervening sequennce (D) or decreased via truncation (E). Indicated reporters were transfected into HeLa cells overexpressing either empty vector or 4E-BP1-4A. In all panels significance was calculated by unpaired, two-sided, t-test adjusted for multiple testing. ns= not significant.

Suppl. Figure 19

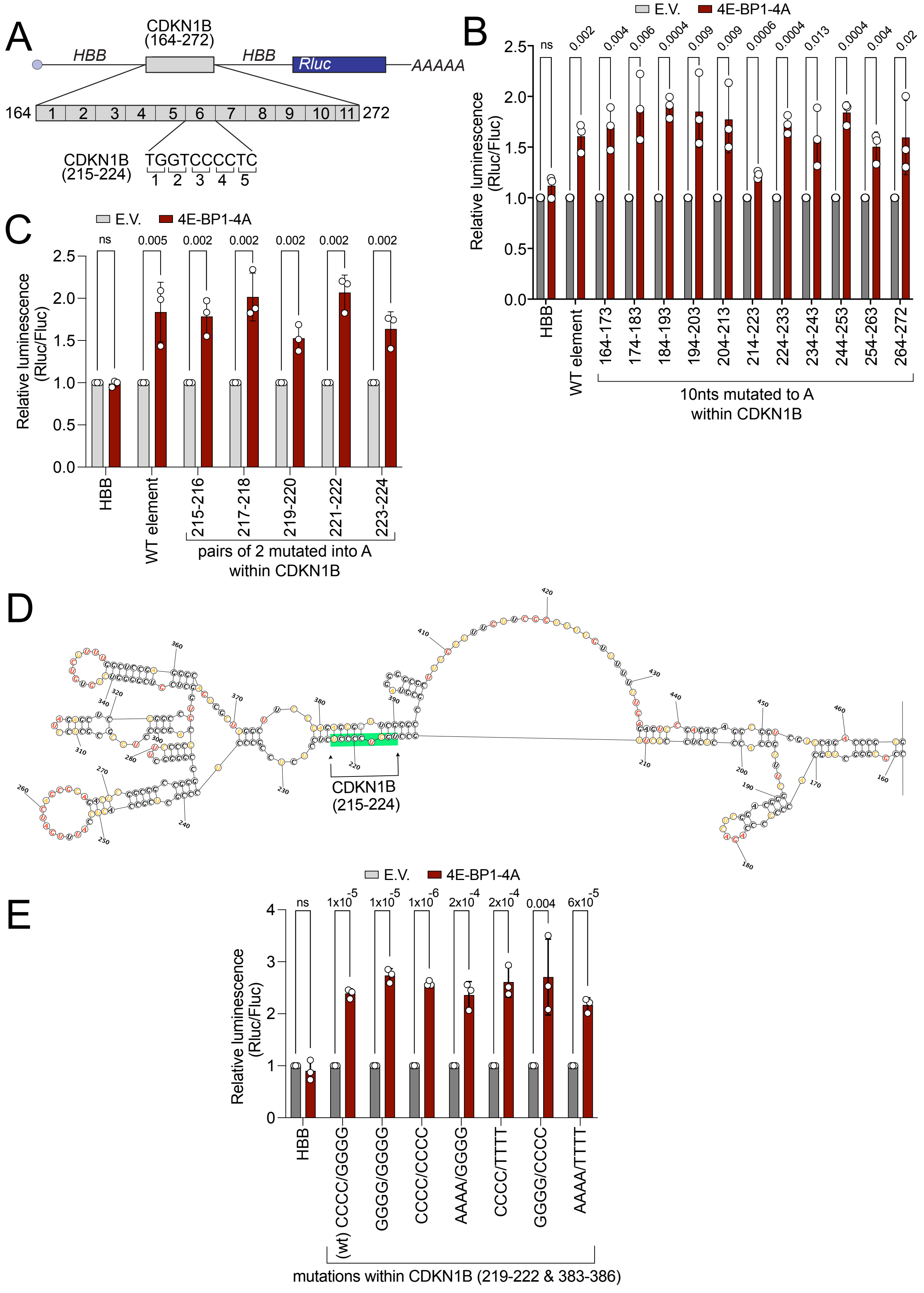

Suppl. Figure 19: Determination of element required for *CDKN1B* resistance.

(A-C) Sequential mutations of nucleotides within the *CDKN1B* functional element identify nucleotides 215-224 as being important region for resistance. (A) Schematic illustration of the *CDKN1B* functional element cloned into the *HBB* 5'UTR of a reporter. Blocks of 10 consecutive nucleotides were mutated to A. The sequence of the 6th block of 10 nucleotides is shown. (B) Indicated reporters carrying adenonsine mutations were transfected into HeLa cells overexpressing either empty vector or 4E-BP1-4A. (C) More detailed mutagenesis was performed by mutating residues 215-224 in blocks of 2 at a time.

(D) Structure of the *CDKN1B* 5' UTR (160-466) defined by SHAPE. Nucleotides identified as crucial in B are highlighted with a green rectangle.

(E) Mutations within the *CDKN1B* functional element which are predicted to disrupt the stem secondary structure do not affect 4E-BP resistance. Residues mutated in the functional element and in the other side of the stem are indicated. Reporters were transfected into HeLa cells overexpressing either empty vector or 4E-BP1-4A.

In all panels significance was calculated by unpaired, two-sided, t-test adjusted for multiple testing. ns= not significant.

(continues on next page)

Uncropped Immunoblots of Supplementary Figures

Suppl. Figure 1

PANEL C

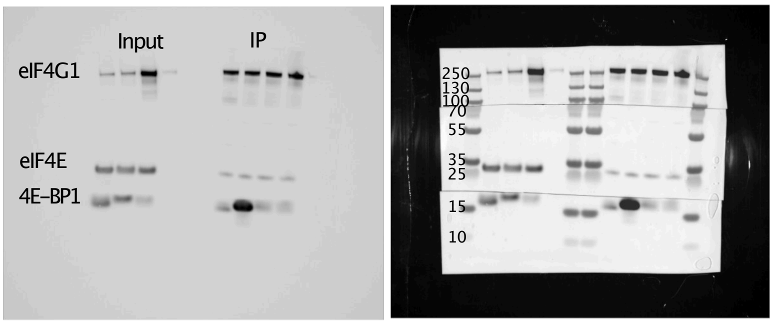

the middle membrane was rebloted with Tubulin antibodies

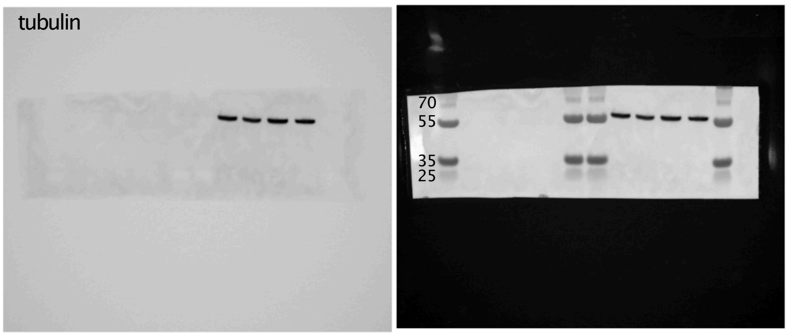

PANEL G

Coomassie blue

puromycin

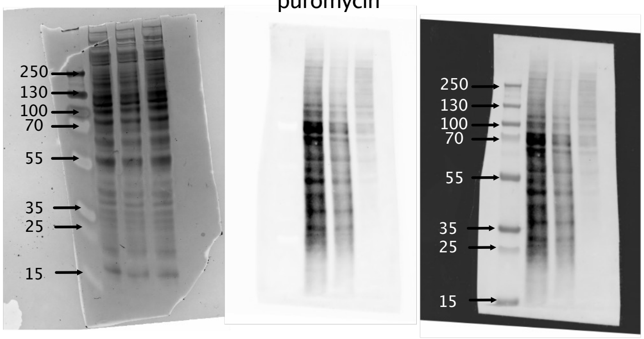

membrane was stripped and rebloted with Tubulin antibodies

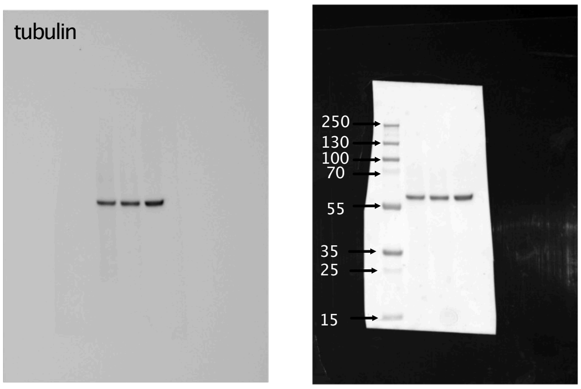

Suppl. Figure 1

PANEL E

Short exposure

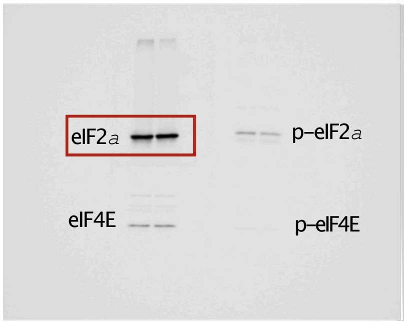

Medium exposure

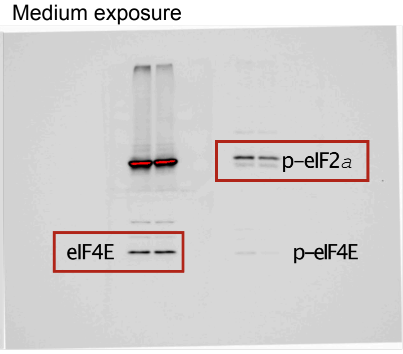

Long exposure

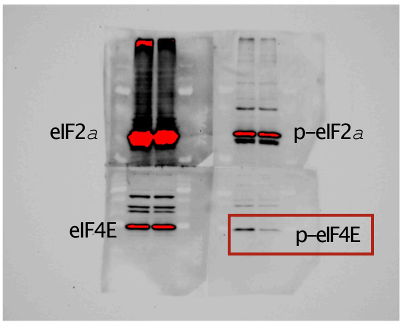

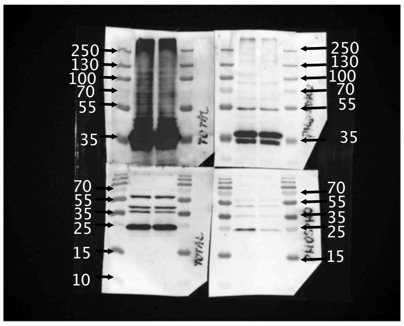

one membrane was rebloted for 4E-BP1

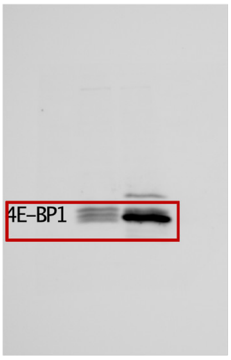

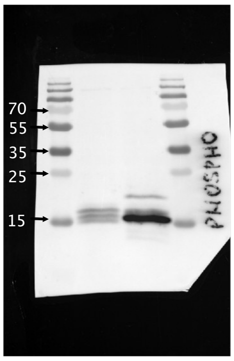

Suppl. Figure 2

PANEL B

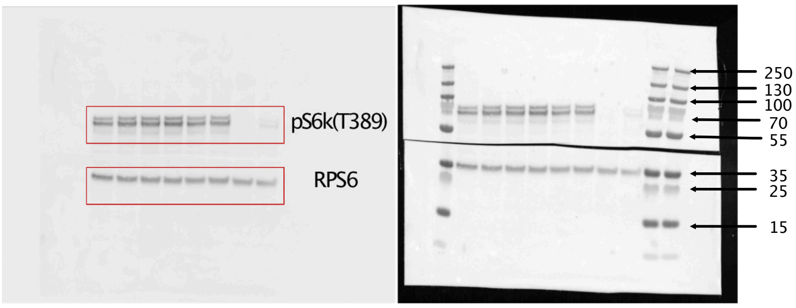

membranes were stripped and re-incubated

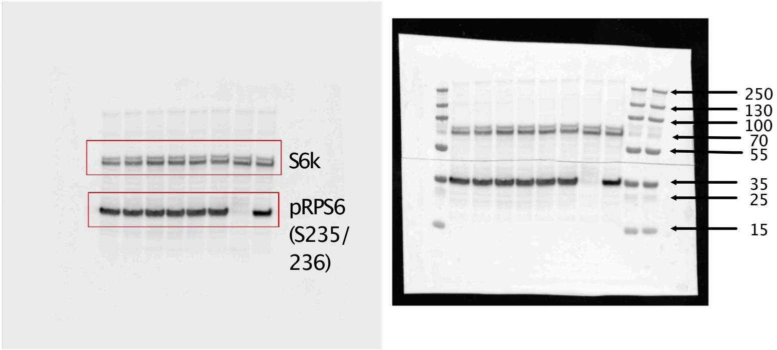

top membrane was stripped and re-incubated against tubulin

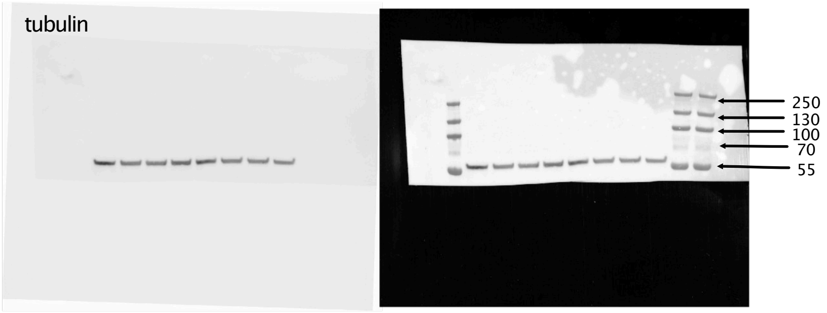

PANEL D

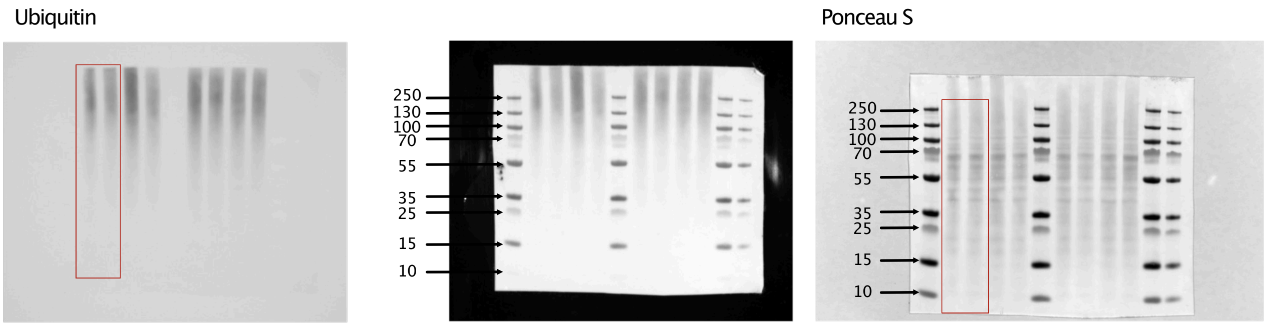

PANEL F

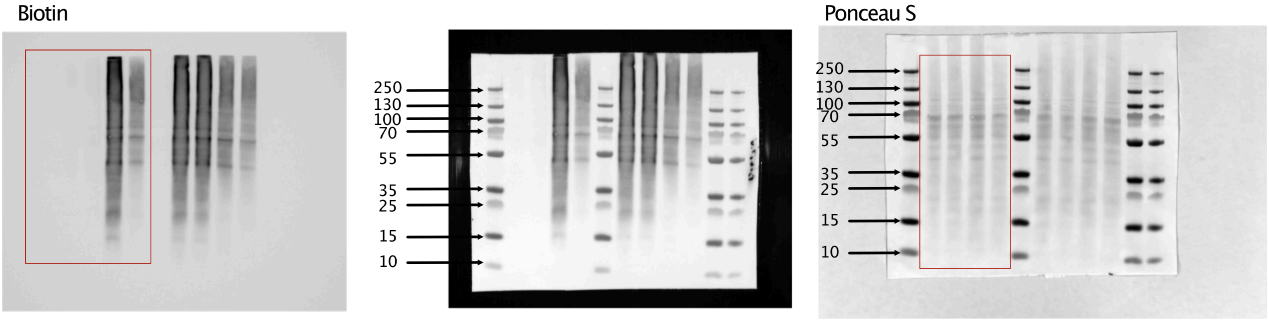

Suppl. Figure 3

PANEL A

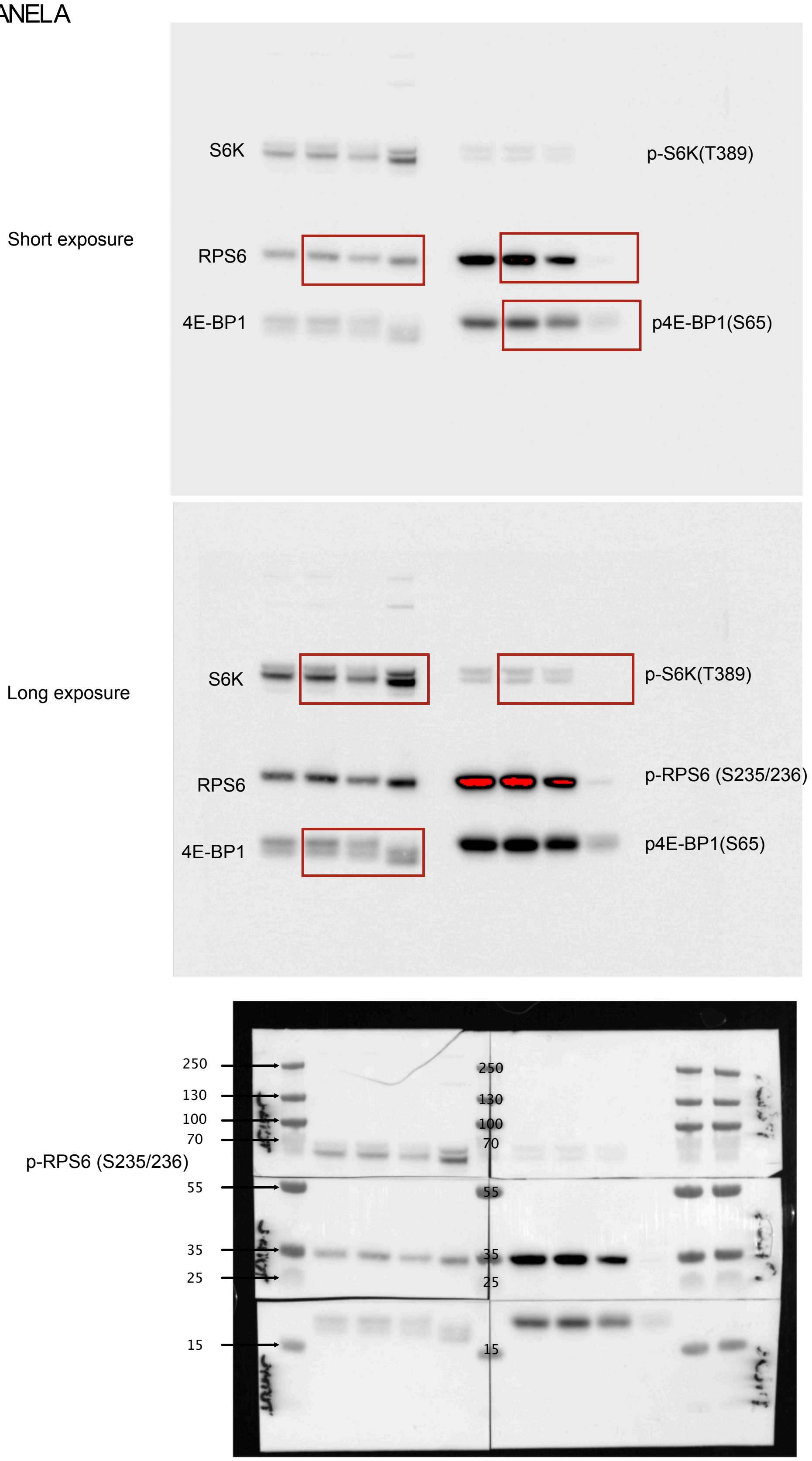

PANEL A

Suppl. Figure 5

eIF4E

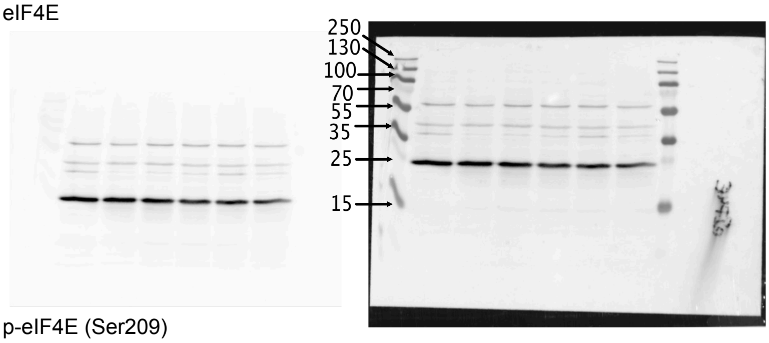

eIF2a

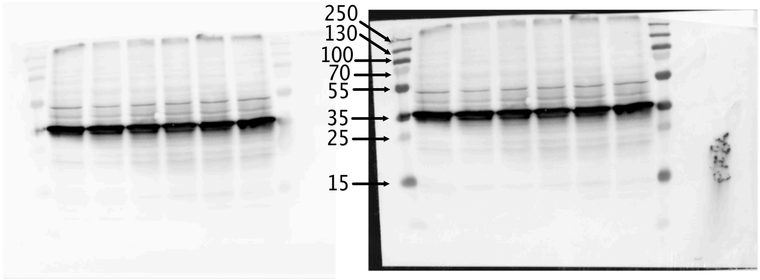

p-eIF4E (Ser209)

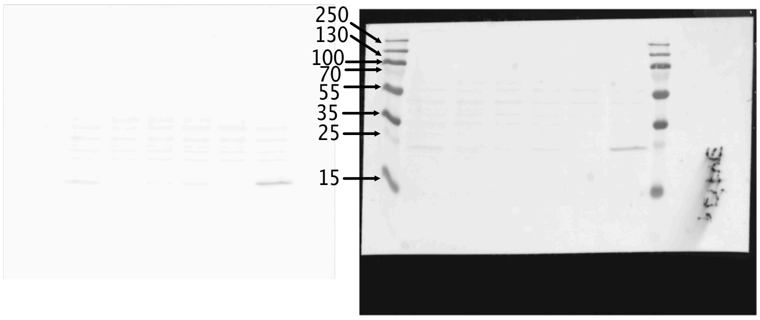

p-eIF2a (Ser51)

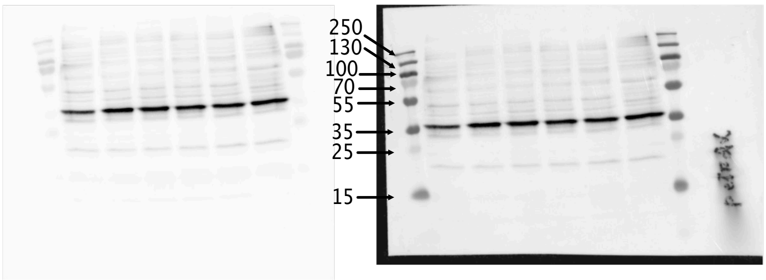

membranes were rebloted with new antibodies

RPS6

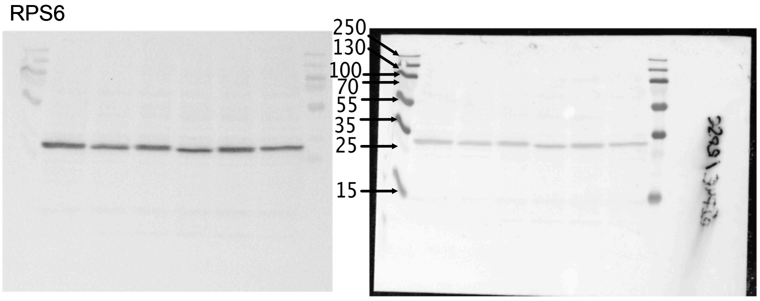

p-RPS6 (S235/236)

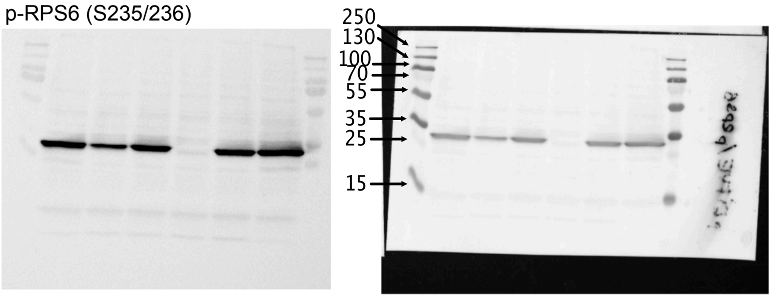

S6K1

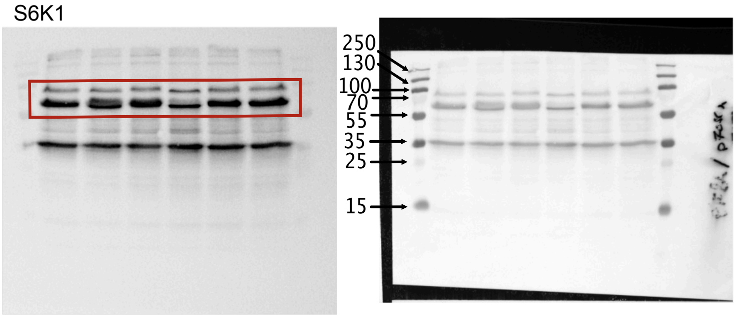

p-T389-S6K1

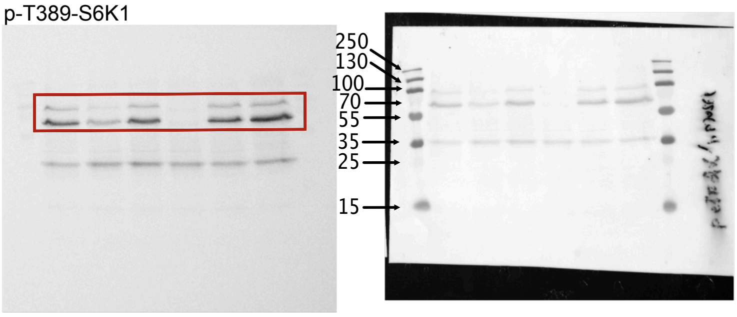

membranes were rebloted with new antibodies

4E-BP1

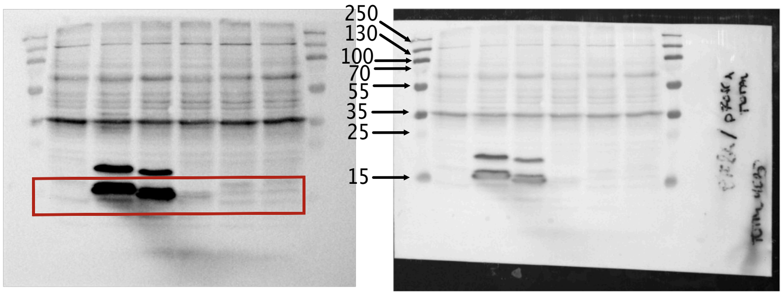

p-4E-BP1(S65)

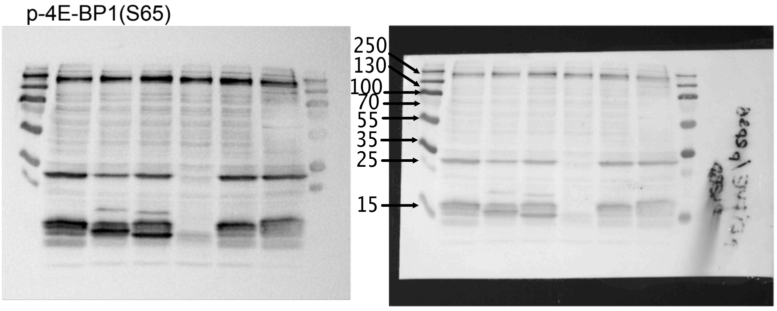

HSP90

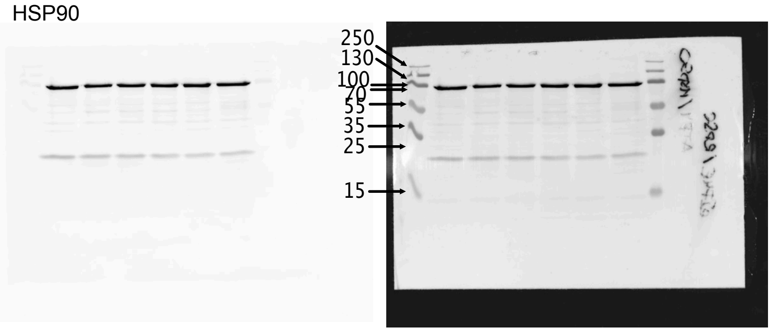

ATF-4

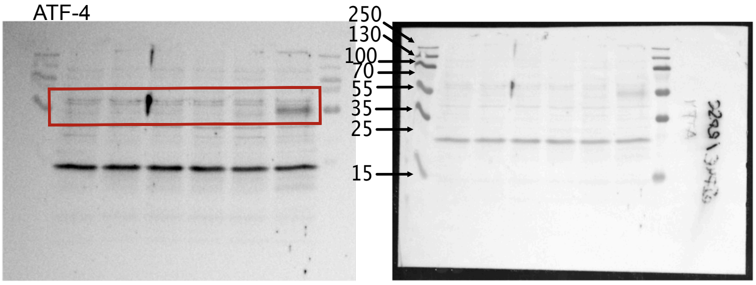

Suppl. Figure 5

PANEL B

short exposure

long exposure

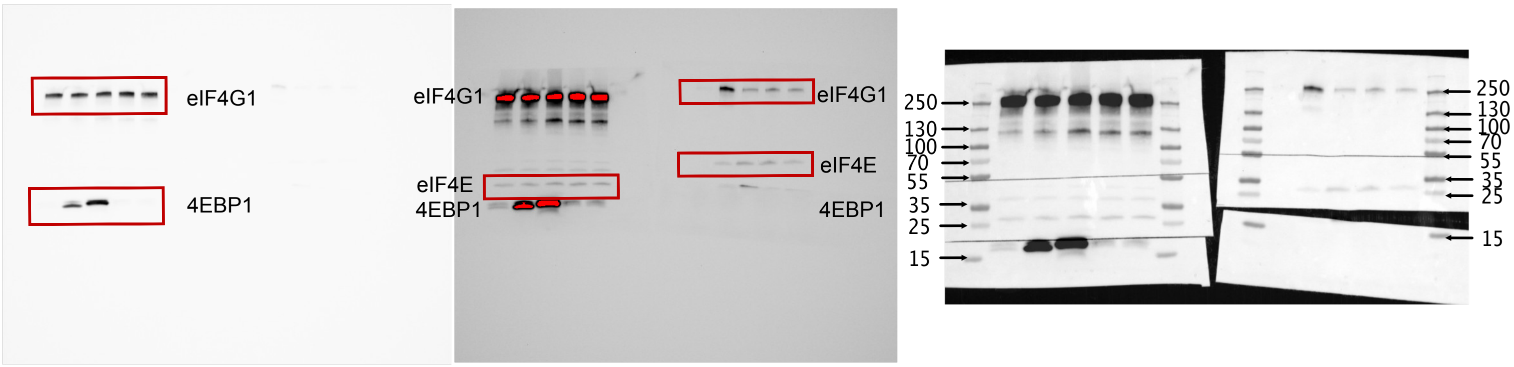

PANEL C

Ponceau S

Pupomycin

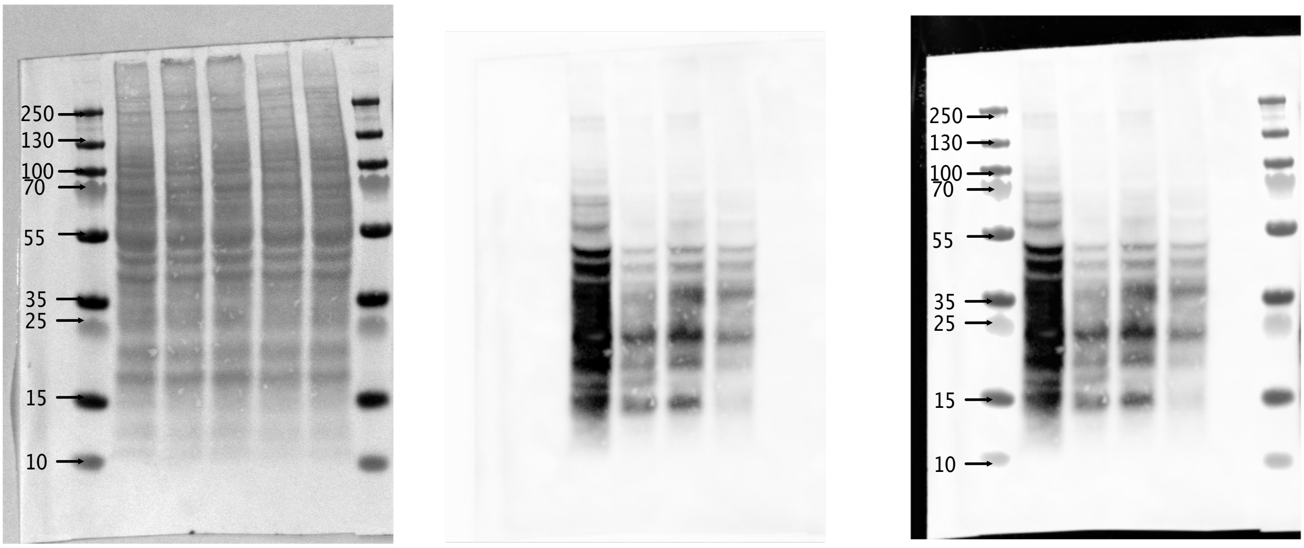

membrane was stripped and rebloted with first 4E-BP1 and then tubulin

4E-BP1

tubulin

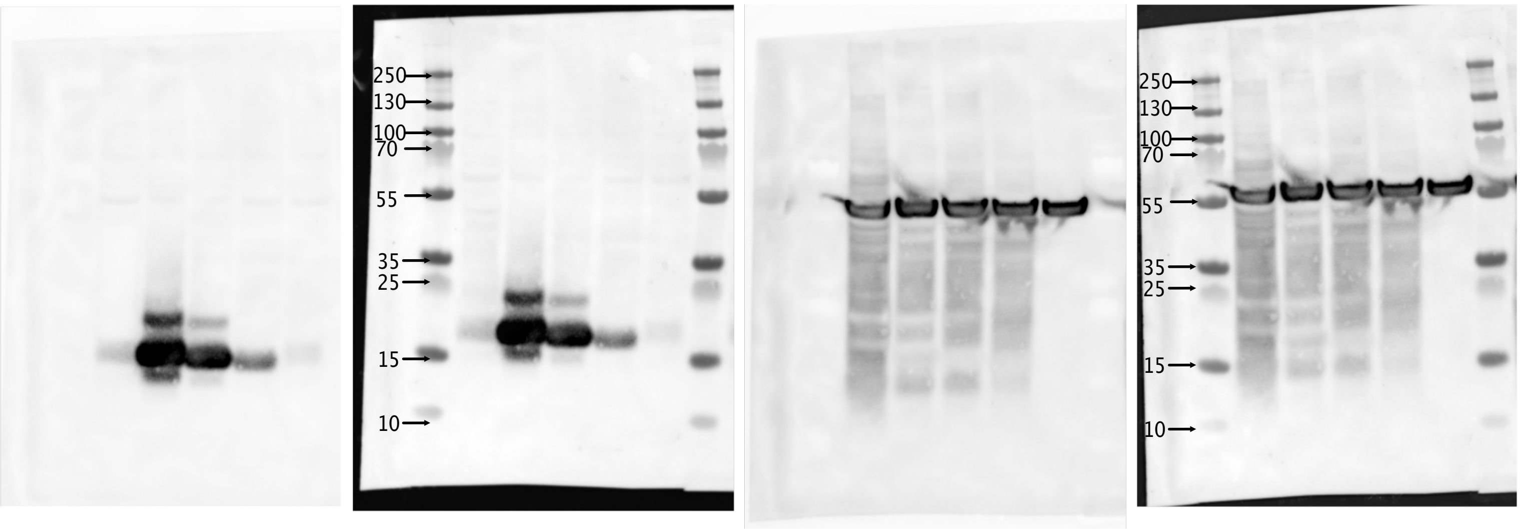

Suppl. Figure 7

PANEL A

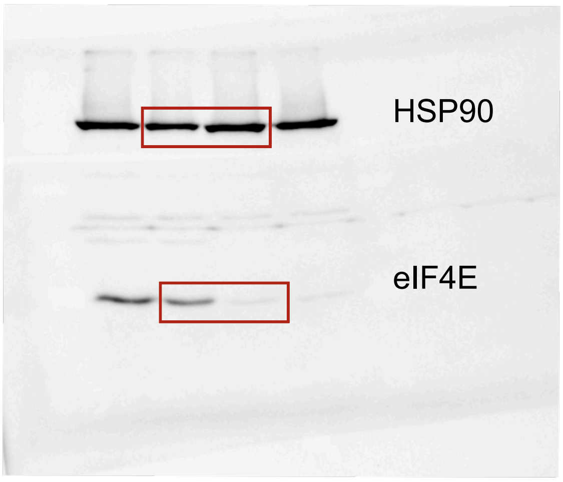

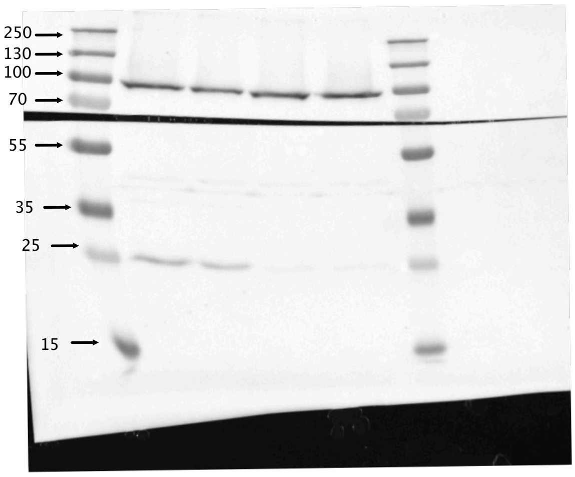

Suppl. Figure 8

PANEL B

membrane was stripped and rebloted with Tubulin antibodies

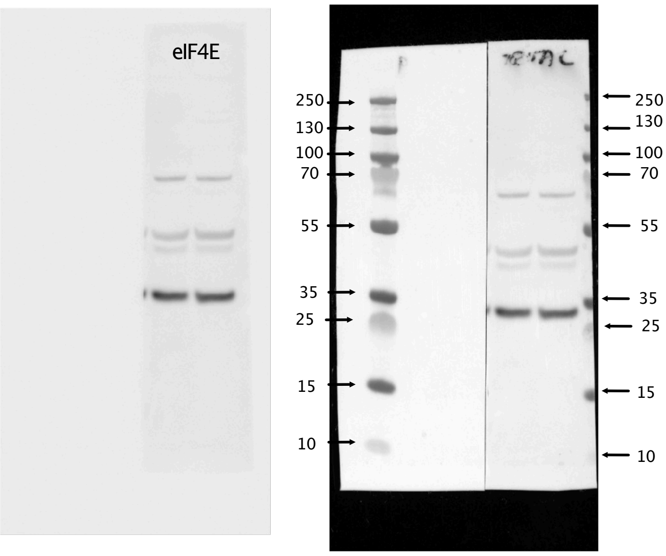

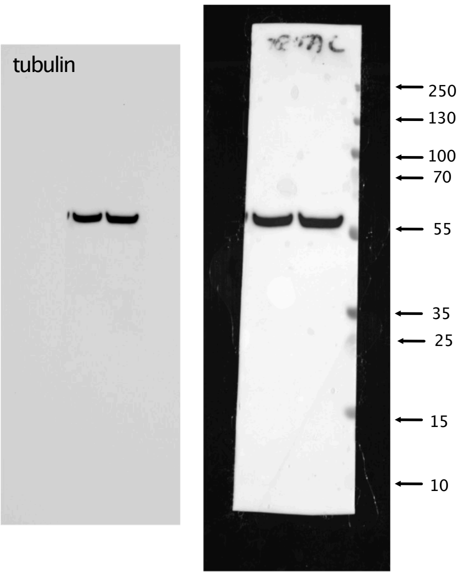

phosphosignal was developed with femto:

p-eIF4E

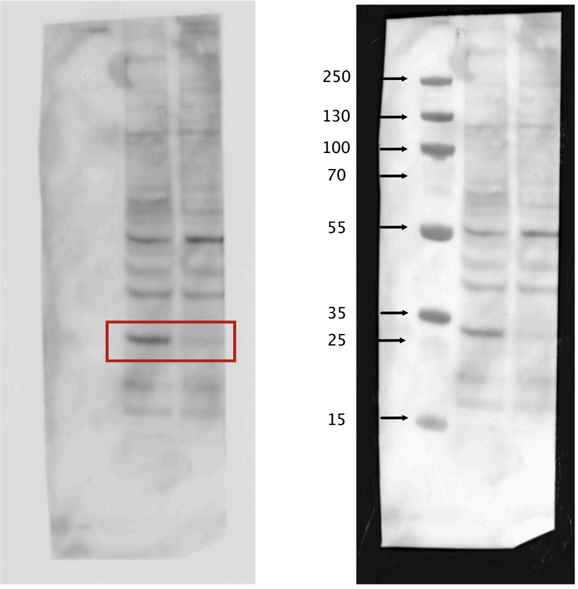

Suppl. Figure 9

PANEL D

m6A

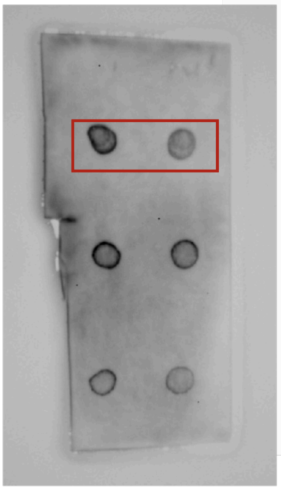

methylene blue

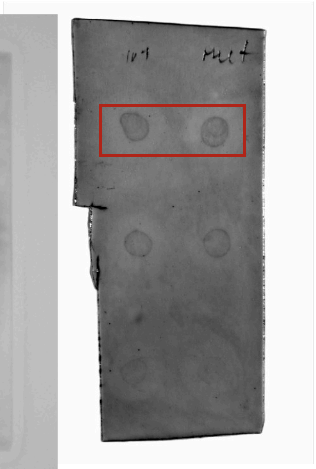

PANEL F

m6A

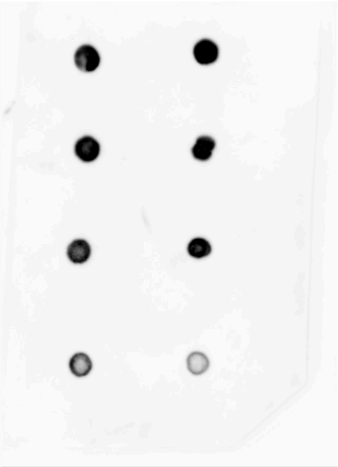

methylene blue

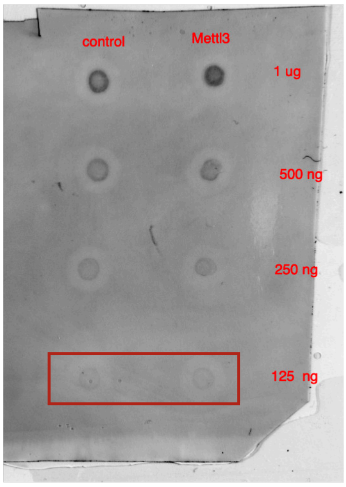

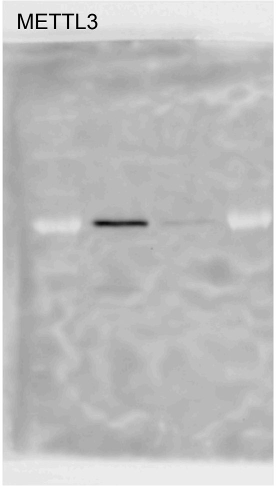

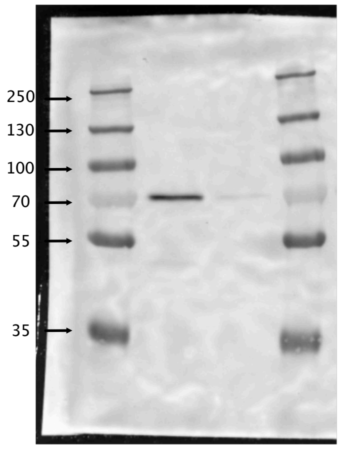

membrane was stripped and rebloted with Tubulin antibodies

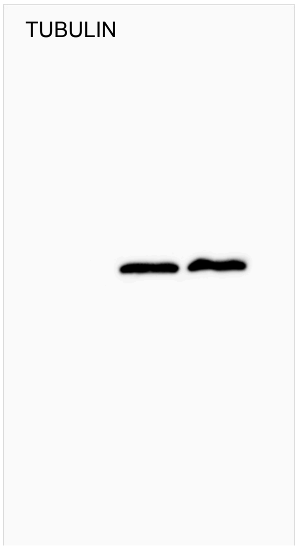

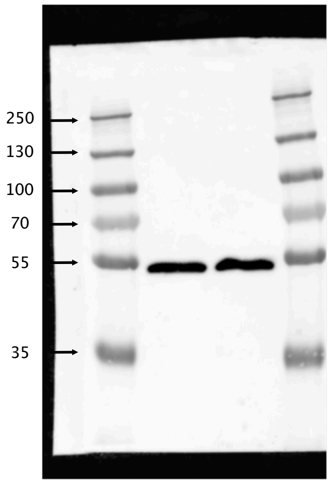

Suppl. Figure 10

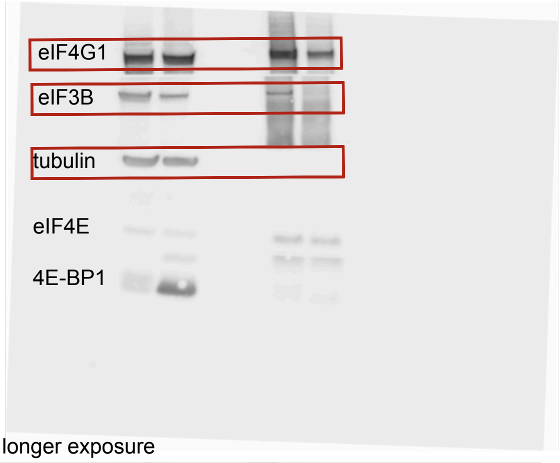

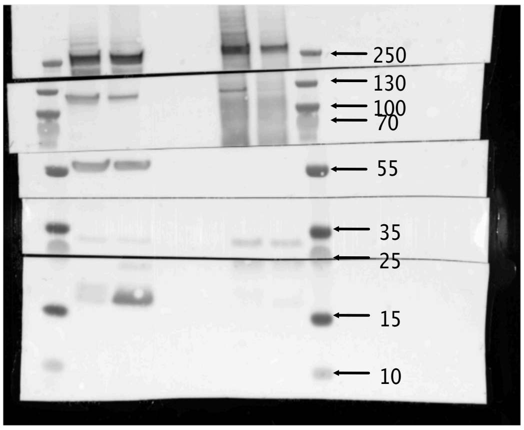

longer exposure

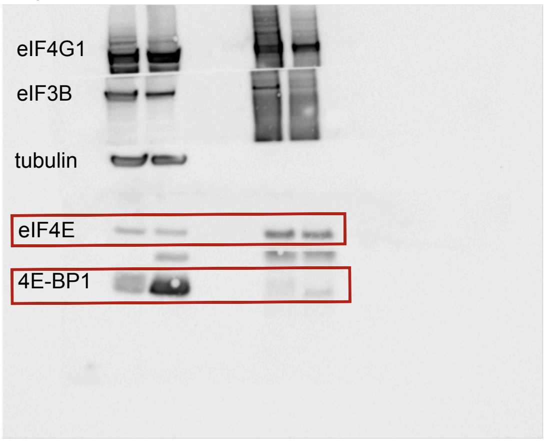

overexposed

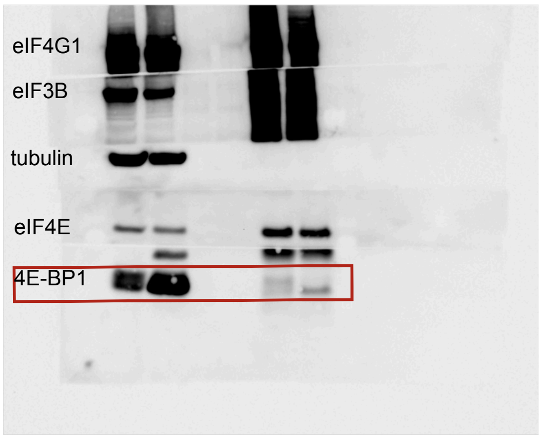

4E-BP1 membrane was stripped and rebloted against RPS15

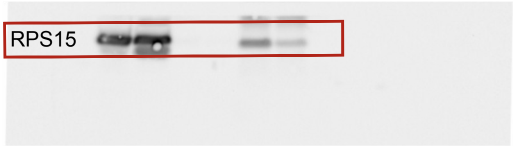

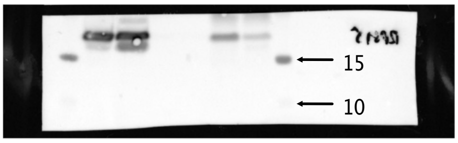

Suppl. Figure 11

PANEL A

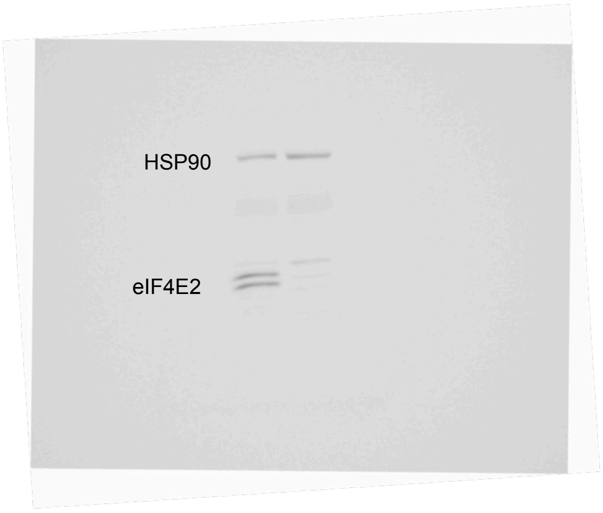

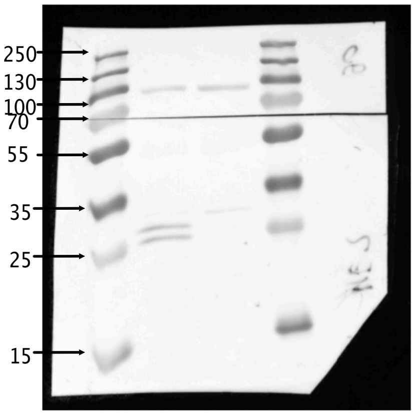

PANEL C

short exposure

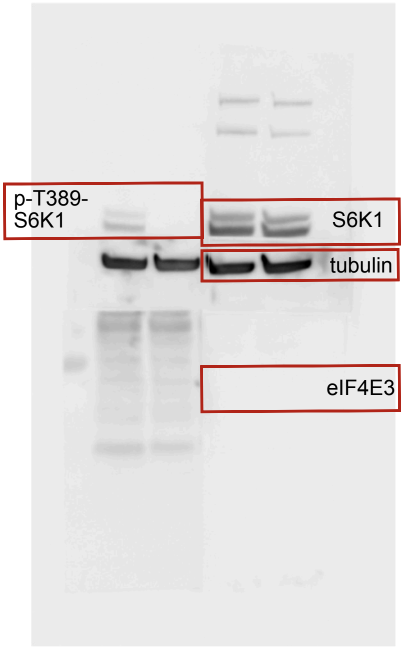

overexposed

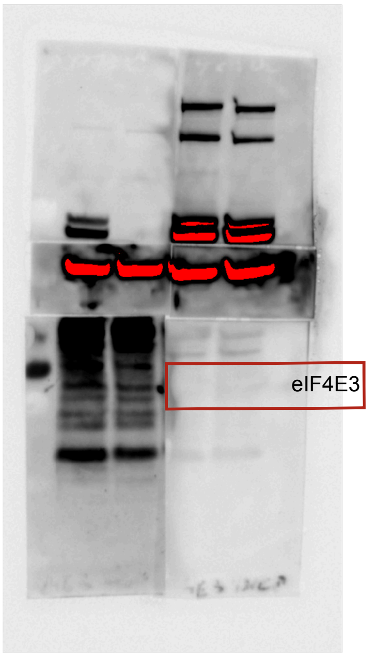

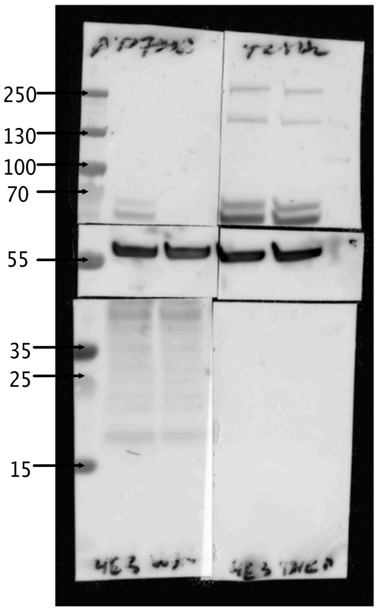

Suppl. Figure 12

PANELA

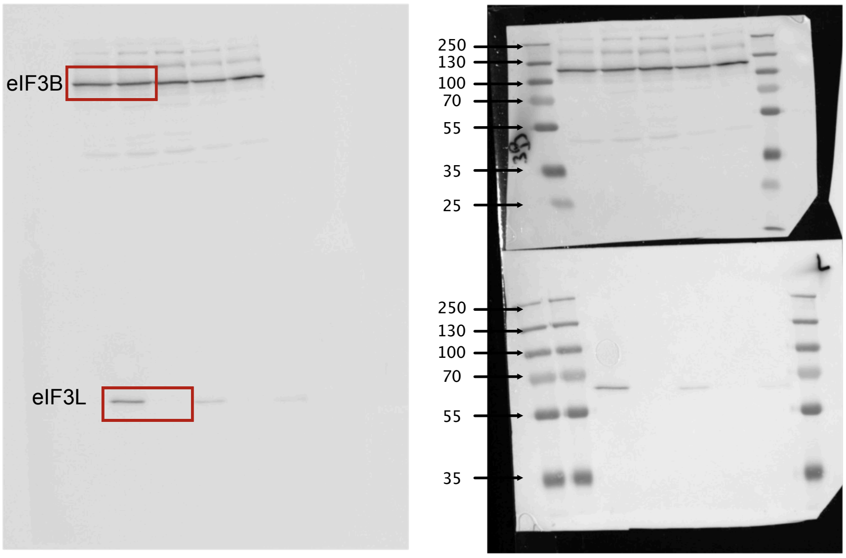

membrane was stripped and rebloted with Tubulin antibodies

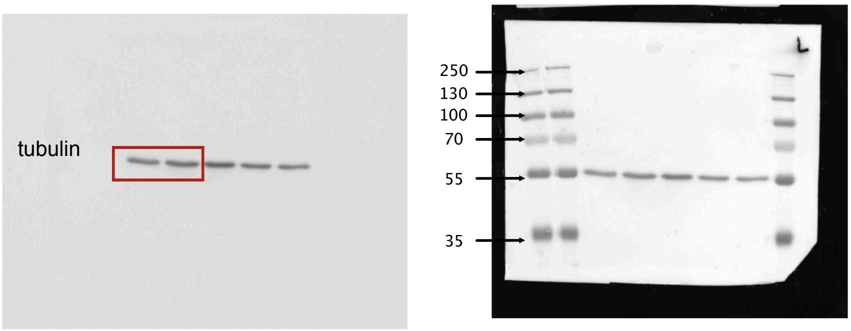

separate gel with same samples was run for eIF3K

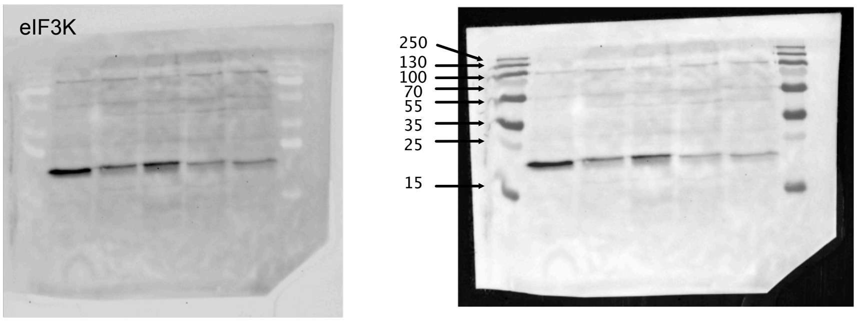

Suppl. Figure 13

PANEL F

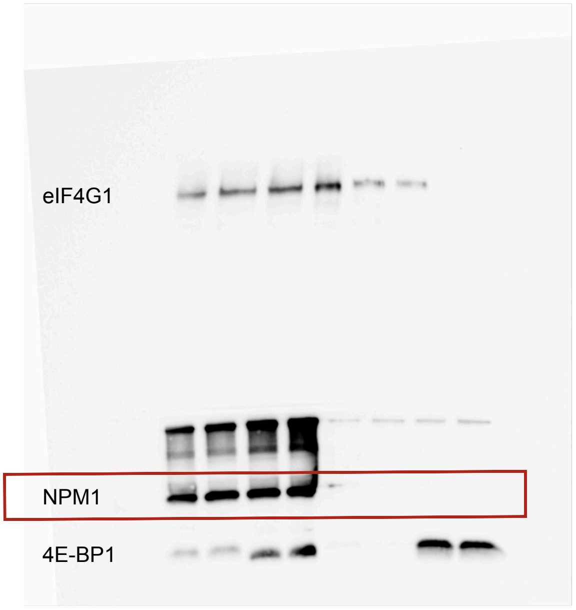

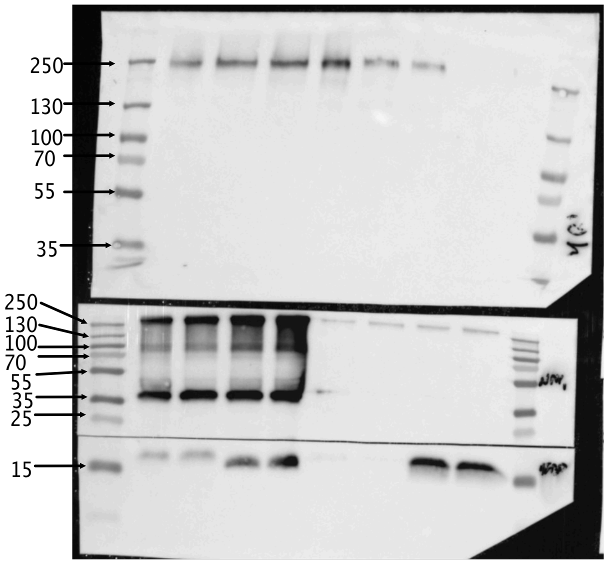

NPM1 membrane was rebloted against eIF4E1

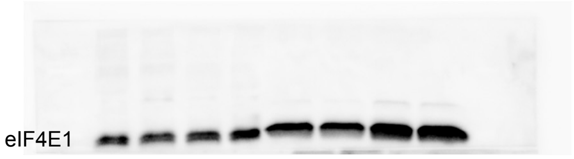

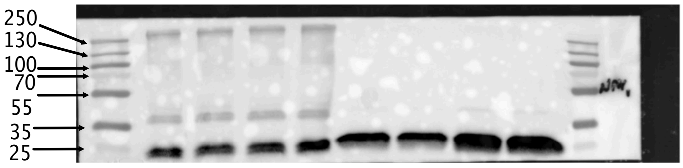

Suppl. Figure 14

PANEL B

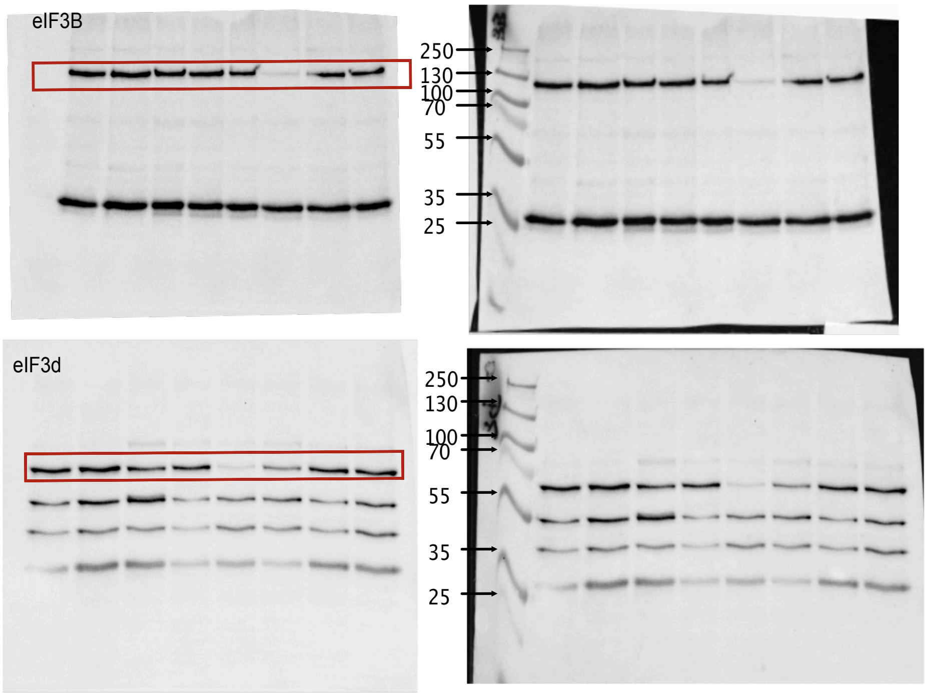

membranes were stripped and rebloted against eIF4G2 and eIF4A1

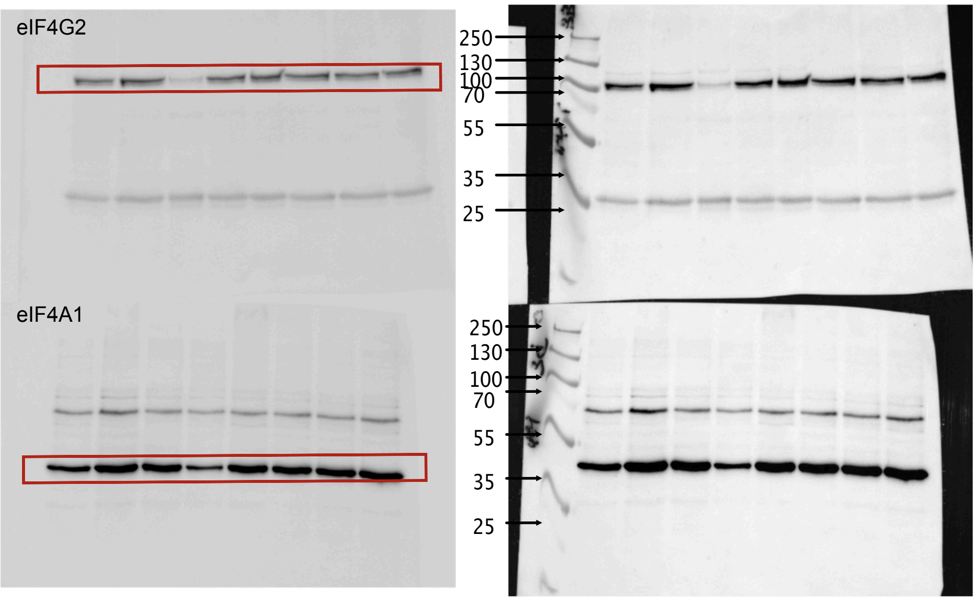

continued on next page

Suppl. Figure 14 (cont.)

PANEL B

continued from previous page

membranes were stripped and rebloted against eIF4E and eIF4A2

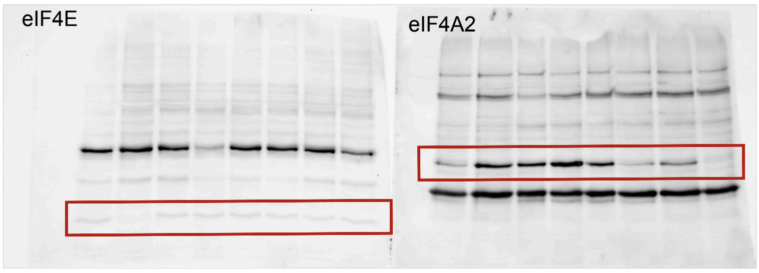

eIF4E (with marker overlay) eIF4A2 (with marker overlay)

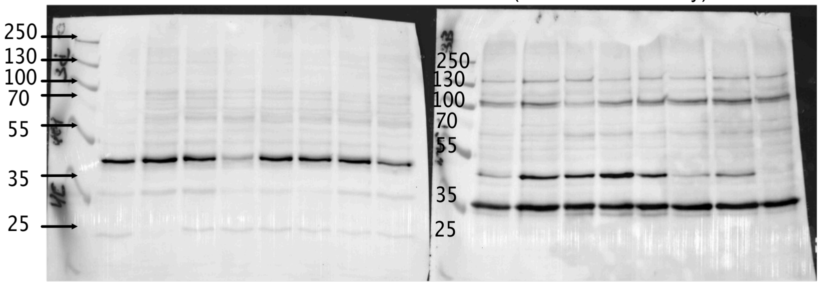

left membrane was stripped and reblotted against eIF4G1

eIF4G1

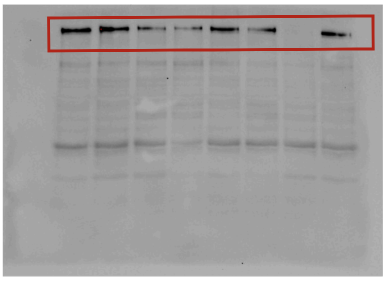

eIF4G1 (with marker overlay)

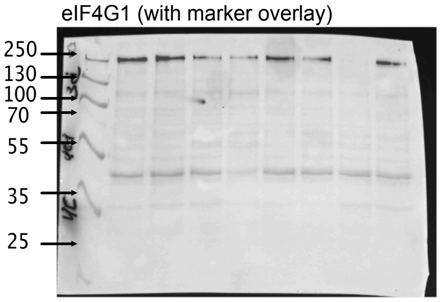

Suppl. Figure 15

PANEL C

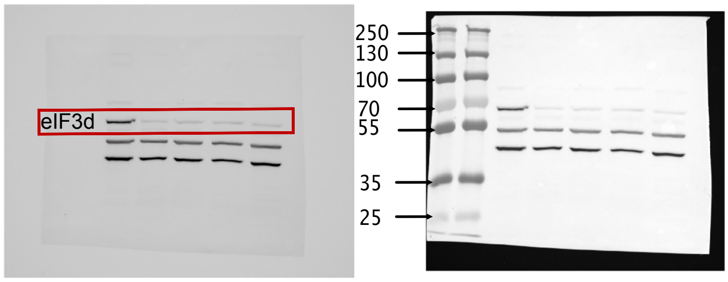

membrane was rebloted against tubulin

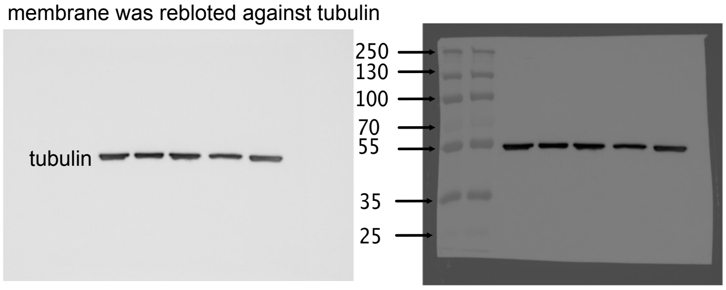

Suppl. Figure 16

PANEL A

ponceau S   biotin

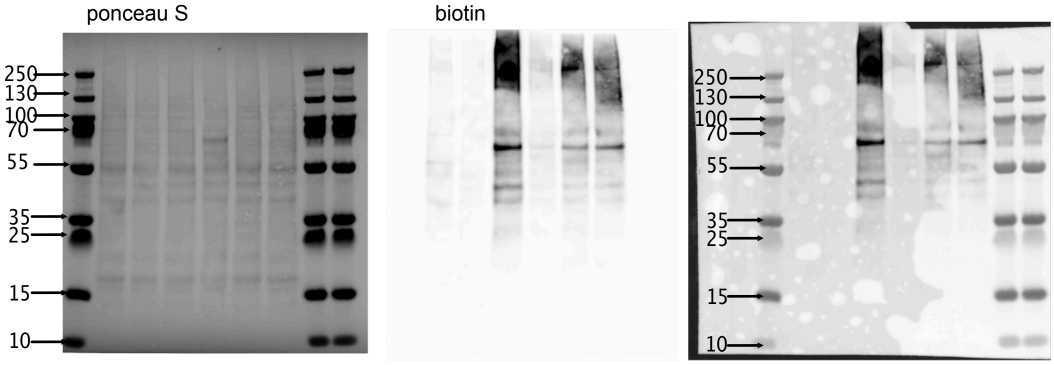

membrane was striped and rebloted against FLAG

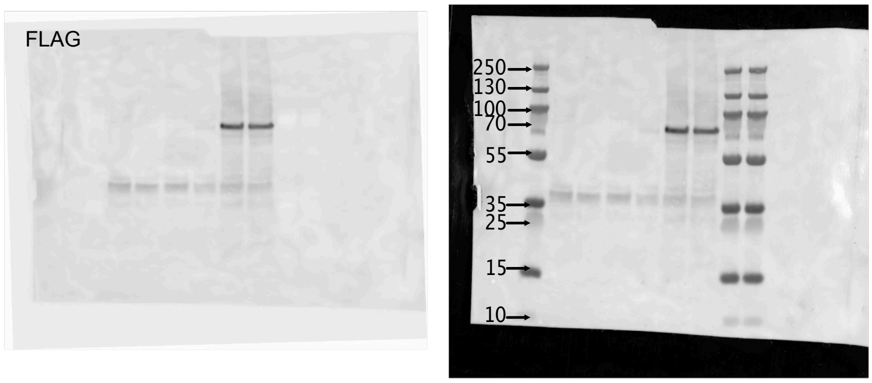

membrane was striped and rebloted against eIF3d

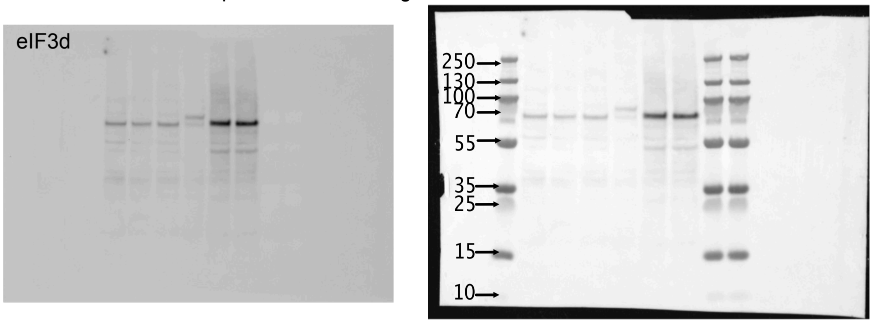

membrane was striped and rebloted against HSP90

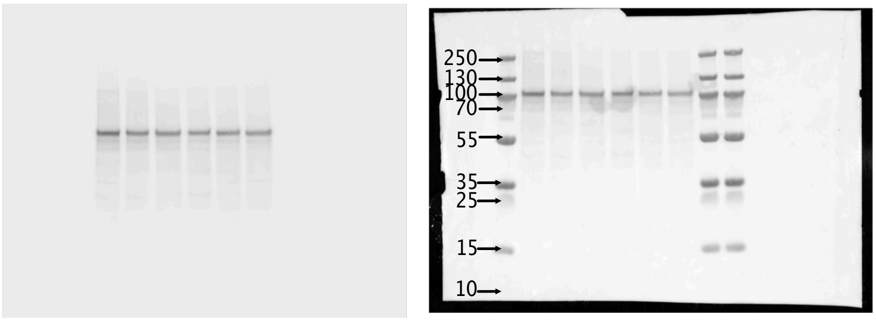

References for Supplementary Information

1. Bekker-Jensen DB*, et al.* An Optimized Shotgun Strategy for the Rapid Generation of Comprehensive Human Proteomes. *Cell Syst* **4**, 587-599 e584 (2017).

2. Zhao W*, et al.* POSTAR3: an updated platform for exploring post-transcriptional regulation coordinated by RNA-binding proteins. *Nucleic Acids Res* **50**, D287-D294 (2022).
