# Supplementary material for: eIF4E-independent translation is largely eIF3d-dependent
Source: Nat Commun. 2024 Aug 6;15:6692. doi: 10.1038/s41467-024-51027-z (PMC11303786; doi:10.1038/s41467-024-51027-z)
Supplement: Supplementary file 3 — Description of Additional Supplementary Files [file 41467_2024_51027_MOESM3_ESM.pdf]

Description of Additional Supplementary Files

Suppl. Data 1: Sequences of sgRNAs used in this study.

Suppl. Data 2: Antibodies used in this study.

Suppl. Data 3: Sequences of oligos used for cloning in this study.

Suppl. Data 4: ENSEMBL transcript IDs for 5'UTRs used for luciferase reporters.

Suppl. Data 5: Sequences of oligos used for Q-RT-PCR in this study.

Suppl. Data 6: siRNAs used in this study

Suppl. Data 7: Effect of knocking out cap-binding proteins on inducibility of the RPP25 reporter upon 4E-BP1-4A expression.

Suppl. Data 8: List of transcripts excuded from the riboseq analysis due to PCR artefacts, detectable as a large accumulation of identical amplicons.
