# Supplementary material for: eIF4E-independent translation is largely eIF3d-dependent
Source: Nat Commun. 2024 Aug 6;15:6692. doi: 10.1038/s41467-024-51027-z (PMC11303786; doi:10.1038/s41467-024-51027-z)
Supplement: Supplementary file 12 — Reporting Summary [file 41467_2024_51027_MOESM12_ESM.pdf]

nature portfolio

Corresponding author(s): Aurelio Teleman

Last updated by author(s): Apr 20, 2024

Reporting Summary

Nature Portfolio wishes to improve the reproducibility of the work that we publish. This form provides structure for consistency and transparency in reporting. For further information on Nature Portfolio policies, see our Editorial Policies and the Editorial Policy Checklist.

Statistics

For all statistical analyses, confirm that the following items are present in the figure legend, table legend, main text, or Methods section.

n/a	Confirmed	
☐	☒	The exact sample size (*n*) for each experimental group/condition, given as a discrete number and unit of measurement
☐	☒	A statement on whether measurements were taken from distinct samples or whether the same sample was measured repeatedly
☐	☒	The statistical test(s) used AND whether they are one- or two-sided<br>*Only common tests should be described solely by name; describe more complex techniques in the Methods section.*
☒	☐	A description of all covariates tested
☐	☒	A description of any assumptions or corrections, such as tests of normality and adjustment for multiple comparisons
☐	☒	A full description of the statistical parameters including central tendency (e.g. means) or other basic estimates (e.g. regression coefficient) AND variation (e.g. standard deviation) or associated estimates of uncertainty (e.g. confidence intervals)
☐	☒	For null hypothesis testing, the test statistic (e.g. *F*, *t*, *r*) with confidence intervals, effect sizes, degrees of freedom and *P* value noted<br>*Give P values as exact values whenever suitable.*
☒	☐	For Bayesian analysis, information on the choice of priors and Markov chain Monte Carlo settings
☒	☐	For hierarchical and complex designs, identification of the appropriate level for tests and full reporting of outcomes
☐	☒	Estimates of effect sizes (e.g. Cohen's *d*, Pearson's *r*), indicating how they were calculated

Our web collection on statistics for biologists contains articles on many of the points above.

Software and code

Policy information about availability of computer code

Data collection	Measurement of luminescence was done with a TriStar Multimode Reader (LB942, Berthold). Chemiluminescence of western blots was recorded with the Chemidoc Imager (Bio-Rad) and quantified using Image Lab software (Bio-Rad).FACS analysis was performed with Guava easyCyte HT (Millipore).
Data analysis	All softwares used in this study are publicly or commercially available: Figure preparation: Affinity Photo Software. Data analysis with Microsoft Excel and GraphPad Prism 9 Software. NGS sequencing data analysis: Bowtie 2 version 2.3.4.2, Cutadapt 2.3 with Python 3.6.4, BBMap version 37.93 and custom software available at github: https://github.com/aurelioteleman/Teleman-Lab

For manuscripts utilizing custom algorithms or software that are central to the research but not yet described in published literature, software must be made available to editors and reviewers. We strongly encourage code deposition in a community repository (e.g. GitHub). See the Nature Portfolio guidelines for submitting code & software for further information.

Data

Policy information about availability of data

All manuscripts must include a data availability statement. This statement should provide the following information, where applicable:

- Accession codes, unique identifiers, or web links for publicly available datasets
- A description of any restrictions on data availability
- For clinical datasets or third party data, please ensure that the statement adheres to our policy

All sequencing data have been deposited at NCBI Geo (GSE243708)

Human research participants

Policy information about studies involving human research participants and Sex and Gender in Research.

Reporting on sex and gender	Not applicable
Population characteristics	Not applicable
Recruitment	Not applicable
Ethics oversight	Not applicable

Note that full information on the approval of the study protocol must also be provided in the manuscript.

Field-specific reporting

Please select the one below that is the best fit for your research. If you are not sure, read the appropriate sections before making your selection.

[x] Life sciences [ ] Behavioural & social sciences [ ] Ecological, evolutionary & environmental sciences

For a reference copy of the document with all sections, see nature.com/documents/nr-reporting-summary-flat.pdf

Life sciences study design

All studies must disclose on these points even when the disclosure is negative.

Sample size	No sample size calculations were performed. All experiments were performed each time on at least 3 times per condition, and the entire experiment was repeated at least 3 times, which from experience is sufficient to assure that the selected examples are representative.
Data exclusions	No replicates were excluded.
Replication	Experiments were replicated at least 3 times. Only data that reproduced are included in this manuscript.
Randomization	All samples were randomized.
Blinding	The experiments were not blinded.

Reporting for specific materials, systems and methods

We require information from authors about some types of materials, experimental systems and methods used in many studies. Here, indicate whether each material, system or method listed is relevant to your study. If you are not sure if a list item applies to your research, read the appropriate section before selecting a response.

Materials & experimental systems

n/a	Involved in the study	
☐	☒	Antibodies
☐	☒	Eukaryotic cell lines
☒	☐	Palaeontology and archaeology
☒	☐	Animals and other organisms
☒	☐	Clinical data
☒	☐	Dual use research of concern

Methods

n/a	Involved in the study	
☒	☐	ChIP-seq
☒	☐	Flow cytometry
☒	☐	MRI-based neuroimaging

Antibodies

Antibodies used	All antibodies used in this study are listed in Suppl. Table 2.
Validation	All antibodies used in the study are are commercially available, they are widely used and are validated on the corresponding webpages of the companies.

Eukaryotic cell lines

Policy information about cell lines and Sex and Gender in Research

Cell line source(s)	HeLa wt, HEK293, U2OS: ATCC
Authentication	The cells were authenticated at Multiplexion using SNP typing.
Mycoplasma contamination	Absence of mycoplasma was confirmed by regular testing of cell cultures (Multiplexion)
Commonly misidentified lines (See ICLAC register)	Not applicable
