# Supplementary material for: eIF4E-independent translation is largely eIF3d-dependent
Source: Nat Commun. 2024 Aug 6;15:6692. doi: 10.1038/s41467-024-51027-z (PMC11303786; doi:10.1038/s41467-024-51027-z)
Supplement: Supplementary file 13 — Source Data [file 41467_2024_51027_MOESM13_ESM.zip › Source data file-1.pdf]

Figure 1

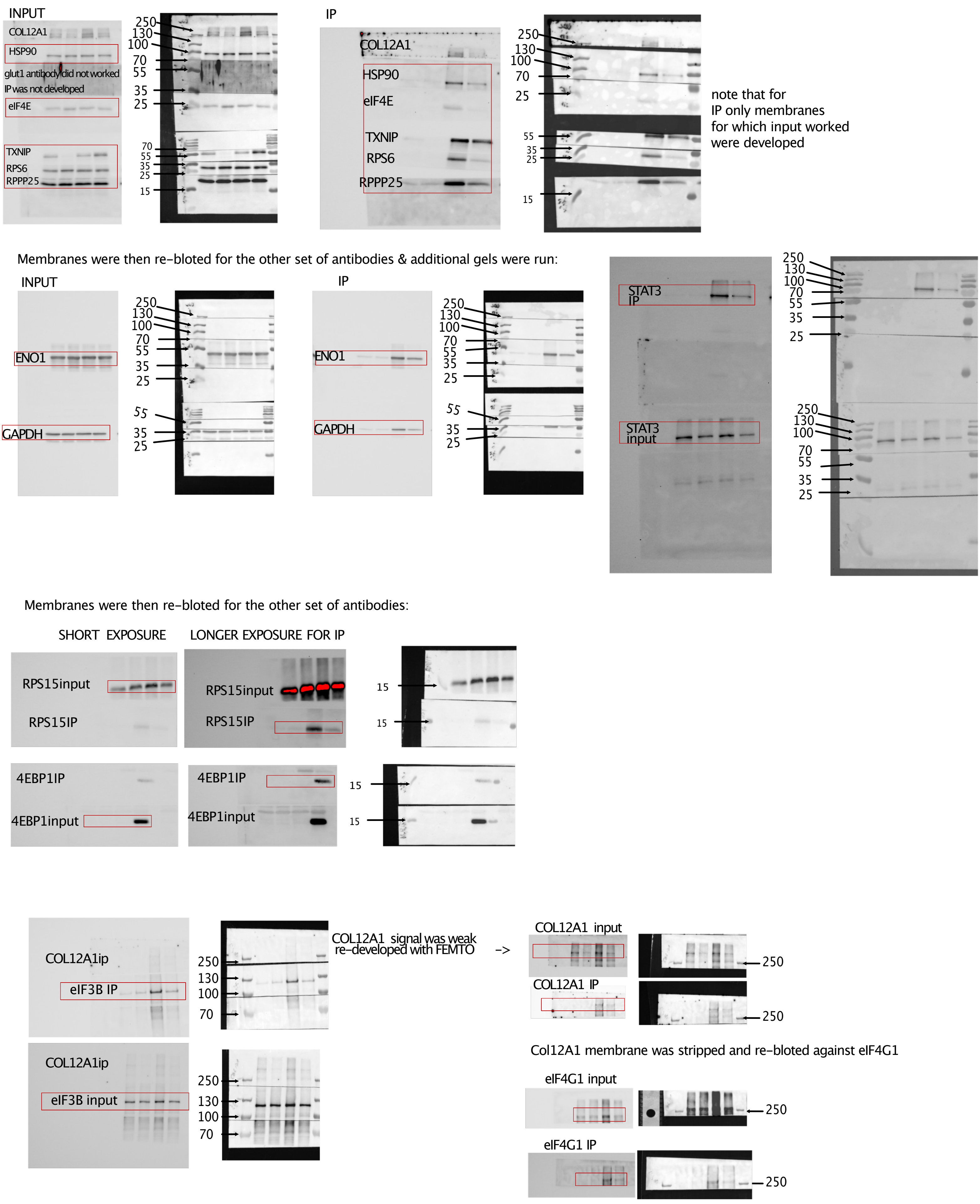

INPUT
COL12A1
HSP90
glut1 antibody did not worked
IP was not developed
eIF4E
TXNIP
RPS6
RPPP25
250
130
100
70
55
35
25
70
55
35
25
15
IP
COL12A1
HSP90
eIF4E
TXNIP
RPS6
RPPP25
250
130
100
70
25
55
35
25
15
note that for
IP only membranes
for which input worked
were developed
Membranes were then re-bloted for the other set of antibodies & additional gels were run:
INPUT
ENO1
GAPDH
250
130
100
70
55
35
25
55
35
25
IP
ENO1
GAPDH
250
130
100
70
55
35
25
55
35
25
STAT3
IP
STAT3
input
250
130
100
70
55
35
25
250
130
100
70
55
35
25
Membranes were then re-bloted for the other set of antibodies:
SHORT EXPOSURE
LONGER EXPOSURE FOR IP
RPS15input
RPS15IP
RPS15input
RPS15IP
15
15
4EBP1IP
4EBP1input
4EBP1IP
4EBP1input
15
15
COL12A1ip
eIF3B IP
250
130
100
70
COL12A1ip
eIF3B input
250
130
100
70
COL12A1 signal was weak
re-developed with FEMTO
->
COL12A1 input
250
COL12A1 IP
250
Col12A1 membrane was stripped and re-bloted against eIF4G1
eIF4G1 input
250
eIF4G1 IP
250
